# Similarities between Exogenously- and Endogenously-Induced Envelope Stress: The Effects of a New Antibacterial Molecule, TPI1609-10

**DOI:** 10.1371/journal.pone.0044896

**Published:** 2012-10-11

**Authors:** Shmuel Yitzhaki, Jason E. Rostron, Yan Xu, Marc C. Rideout, R. Nathan Authement, Steven B. Barlow, Anca M. Segall

**Affiliations:** 1 Department of Biology and Center for Microbial Sciences, San Diego State University, San Diego, California, United States of America; 2 Electron Microscopy Facility, San Diego State University, San Diego, California, United States of America; University of Massachusetts, United States of America

## Abstract

Antibiotics with novel and/or multiple targets are highly desirable in the face of the steady rise of clinical antibiotic resistance. We have screened and identified small molecules, typified by the compound TPI1609-10 (aka SM10), with antibiotic activity against both gram-positive and gram-negative bacteria. SM10 was screened *in vitro* to bind branched Holliday junction intermediates of homologous recombination and tyrosine recombinase-mediated recombination; thus, the cellular targets of the small molecules were expected to include the RuvABC Holliday junction resolvasome and the XerCD complex involved in proper segregation of replicated chromosomes to daughter cells. SM10 indeed induces DNA damage and filamentation in *E. coli*. However, SM10 also induces envelope stress and causes increased production of intracellular reactive oxygen species. In addition, SM10 has similar effects to endogenously-induced envelope stress via overproducing outer membrane proteins (OmpC and OmpF), which also induces the SOS response, chromosome fragmentation, and production of reactive oxygen species. The synergy between SM10, and cerulenin, a fatty acid synthesis inhibitor, together with the SM10 hypersensitivity of *cpx* and *rpoE* mutants, further support that SM10's mode of action damages membrane damage. The lethality of SM10 treatment and of OmpC overproduction are observed in both aerobically- and anaerobically-grown cells, and is accompanied by substantial DNA damage even anaerobically. Thus, only some DNA damage is due to reactive oxygen. We propose that membrane depolarization and the potential reduction in intracellular pH, leading to abasic site formation, cause a substantial amount of the DNA damage associated with both SM10 treatment and endogenous envelope stress. While it is difficult to completely exclude effects related to envelope damage as the sources of DNA damage, trapping intermediates associated with DNA repair and chromosome segregation pathways remains very likely. Thus SM10 may have distinct but synergistic modes of action.

## Introduction

During their lifetime, bacteria may face many environmental challenges in the form of toxic chemicals (naturally occurring or man-made) or physical conditions (suboptimal pH, desiccation, UV or other irradiation, etc.). Internal stresses in the form of reactive oxygen species are also damaging [1]. Different stress responses have evolved to mitigate these challenges, including the SOS response [2], heat shock response [3], acid stress response [4], starvation response [5] and the envelope stress response [6], [7]. Envelope stress guards the integrity of the cell's membranes and thus of the cell itself, and is mediated through the sigma factor σE [6]–[9] and the two component signal transduction systems CpxA/R [6], [10]–[12] and BaeR/S [13]–[15], which respond to both unique and overlapping signals. These three cross–regulate factors (e.g., proteases and chaperones) that protect and restore the integrity of the bacterial envelope [7], [10], [16]. While the envelope stress response was discovered as the means to repair damage due to over-expression of major bacterial porins [17], more recently it has been implicated during bacterial growth in the presence of antibiotics [18]–[21].

Stress responses do not operate in isolation of each other. Multiple stress responses may be invoked, at least in some circumstances. For example, disrupting peptidoglycan synthesis by treatment with ampicillin induces the SOS response in both *E. coli* and *S. aureus* [22], [23]. Sub–lethal physical stress in the form of high pressure (100 MPa) also induces the SOS response [24], although the nature of the inducing signal is still not understood. Fang and colleagues have documented that acid stress also induces envelope stress responses [25], while Kohanski and colleagues have championed the controversial model that treating bacteria with antibiotics such as ampicillin, nalidixic acid, or aminoglycosides such as kanamycin, generates reactive oxygen species (ROS) and induces the SOS response [26], [27]. In the case of the aminoglycosides, mistranslation of membrane proteins was proposed to induce the envelope stress response [27]. Thus a number of stresses appear to induce DNA damage and the SOS response.

**Figure 1:**
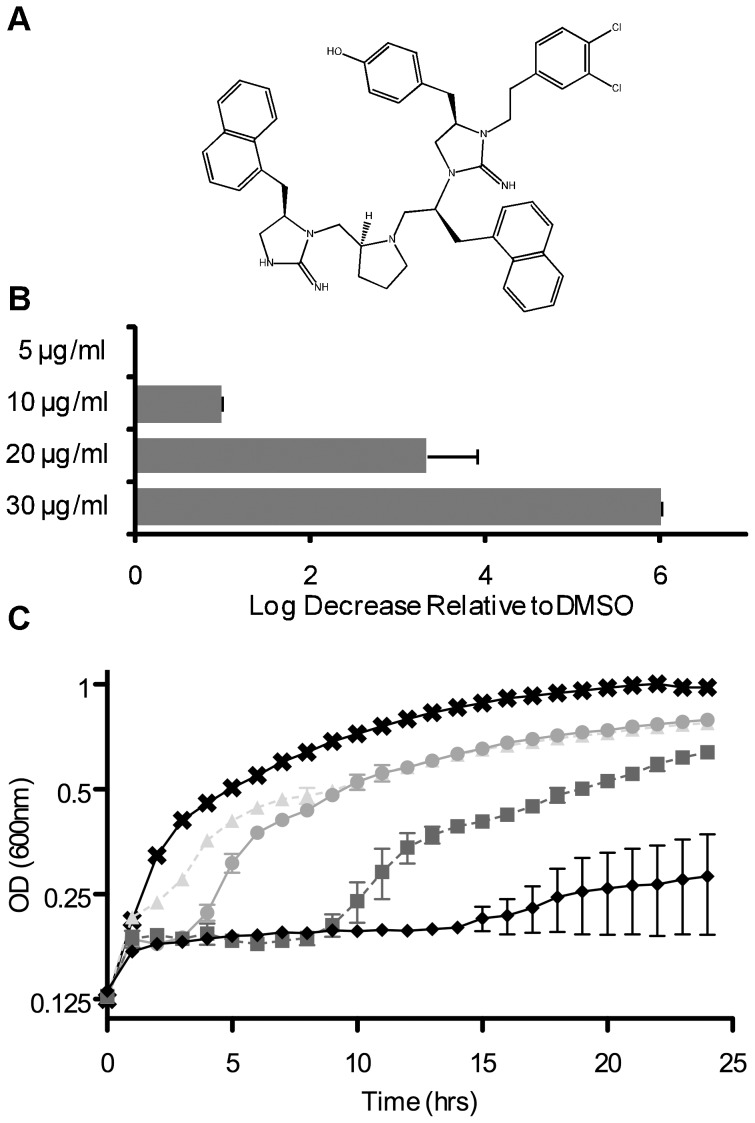
SM10, a synthetic small molecule with antibacterial activity. (**A**) Chemical structure of SM10. (**B**) SM10 causes a dose-dependent drop in *E. coli* MG1655 cell viability. MG1655 cells were incubated in the presence of SM10 in MHB at 37°C for 3 hr, then the cultures were diluted and plated on LB. (**C**) Cells were grown in MHB to an OD_600_ of 0.1, and then treated with different doses of SM10 or with DMSO, as one test for lysis. In panel **C**, the symbols denote the following treatments: **x**'s, DMSO; **triangles**, 5 µg/ml SM10; **circles**, 10 µg/ml SM10; **squares**, 20 µg/ml SM10; and **diamonds**, 30 µg/ml SM10 final concentration.

We have identified a number of related short peptides that bind branched DNA intermediates of recombination and DNA repair, Holliday junctions with highest affinity, and prevent their resolution by junction resolvases and tyrosine recombinases and the related type IB topoisomerases [28]–[33]. Peptides WRWYCR and wrwycr are bactericidal in both Gram-positive and Gram-negative bacteria, cause chromosome segregation defects, and cause accumulation of DNA breaks ([34]; Gunderson and Segall, ms. in prep). These and related peptides stabilize Holliday junction intermediates in bacteria of either phage lambda recombination [35] or homologous recombination-dependent DNA repair (Marcusson, Medina-Cleghorn, Gutierrez, Agrawal, & A.M.S., ms. in prep.). More recently, non-peptide small molecules with similar activities were identified [36], [37]. Small molecule TPI1609-10 (referred to here as SM10), shown in in Figure 1A, was synthesized based on screens of a collection of small molecule libraries for its ability to trap Holliday junction intermediates of phage lambda site-specific recombination (IC_50_ = 1.8 µg/ml). SM10 binds protein-free HJ, although with lesser affinity and/or stability than peptide WRWYCR, wrwycr, or KWWCRW, and inhibits junction resolution by the RuvABC resolvasome and by RecG helicase [37]. Despite this, SM10 has more potent antibiotic activity than the peptides, inhibiting *B. subtilis* and MRSA with an MIC of 2–4 µg/ml, at least 4× lower than the peptides, and *E. coli* and *Salmonella enterica* LT2 with an MIC of 16–32 µg/ml, about 2× lower than the peptides. A differential in MIC values between the small molecules and the peptide was also observed in the hyperpermeable *Salmonella galE rfa* strain (used in the Ames tests for mutagenic and teratogenic potential) [38], suggesting that permeability is not the sole reason for the difference in the MIC of these compounds. This leaves open the possibility that the small molecules affect additional targets.

Despite many attempts, we have been unsuccessful in identifying mutants of *E. coli* or Salmonella resistant to SM10. During our investigation of its mechanism of action, we discovered that SM10 induces both envelope stress and DNA damage, and have demonstrated directly that envelope stress-inducing conditions such as overexpression of porins and treatment with EtOH or indole also induce DNA damage. This is independent of the presence of oxygen during growth: at least 50% of DNA damage occurs anaerobically as well. The similarities and differences between the consequences of porin overexpression and treatment of bacteria with SM10 have important implications for our views of bacterial responses to antibiotic stress and environmental conditions.

## Results

### Synthetic small molecules with antibacterial activity

Several small molecules identified in the same manner as the DNA repair inhibitory peptides, have higher antimicrobial activity than the peptide inhibitors [37]. SM10 was chosen as a prototype for further characterization, and its chemical structure is depicted in Figure 1A. To further characterize the antimicrobial mechanism of SM10, we studied its effect on the viability of *E. coli* MG1655 and showed that it inhibits growth severely at 20–30 µg/ml, and is bactericidal at 30 µg/ml based on a reduction in viability by more than 99.9% compared to starting cell counts (Figure 1B). To test whether SM10 induced cell lysis, we added SM10 to cells 1 hour after sub-culturing *E.coli* in fresh media and followed the optical density at 600 nm (OD_600_) of the culture. While SM10 slowed further growth, it did not reduce the OD_600_ of the cultures (Figure 1C). The recovery of cultures after several hours may be due to the induction of efflux pumps, as seen in the case of peptide wrwycr (Orchard *et al*, 2011; Naili, Rostron, Segall, ms in prep). To test the general effect of media composition on sensitivity to SM10, we compared susceptibility in rich and minimal media and found that *E.coli* MG1655 is even more sensitive to SM10 treatment in minimal media (Figure S1). It is possible that peptides or other constituents of complex media compete with the uptake of SM10.

### SM10 effects on macromolecular syntheses

We performed metabolite incorporation assays to determine whether SM10 affects the synthesis of macromolecules in *E.coli*. The incorporation of ^3^H-labeled thymidine into DNA was not inhibited by SM10 (Figure S2A). Incorporation of ^3^H-labeled uridine and leucine into RNA and proteins, respectively, were slightly inhibited by SM10 at 20 µg/ml (Figure S2B & S2C), which may, at the 30 min time point, reflect inhibition of cell growth by SM10. In contrast to the other metabolites, incorporation of ^14^C-labeled acetate into phospholipids increased slightly with SM10 treatment (Figure S2D). When we repeated the phospholipid incorporation assay, with ^3^H-acetate using low concentrations of SM10 (5 and 10 µg/ml), we saw increases in acetate incorporation of 31.5% and 62.2%, respectively, after 30 min incubation. The results of the radiolabeling experiments suggested that inhibiting DNA, RNA, protein, or phospholipid synthesis is not the primary mechanism of action of SM10.

**Figure 2:**
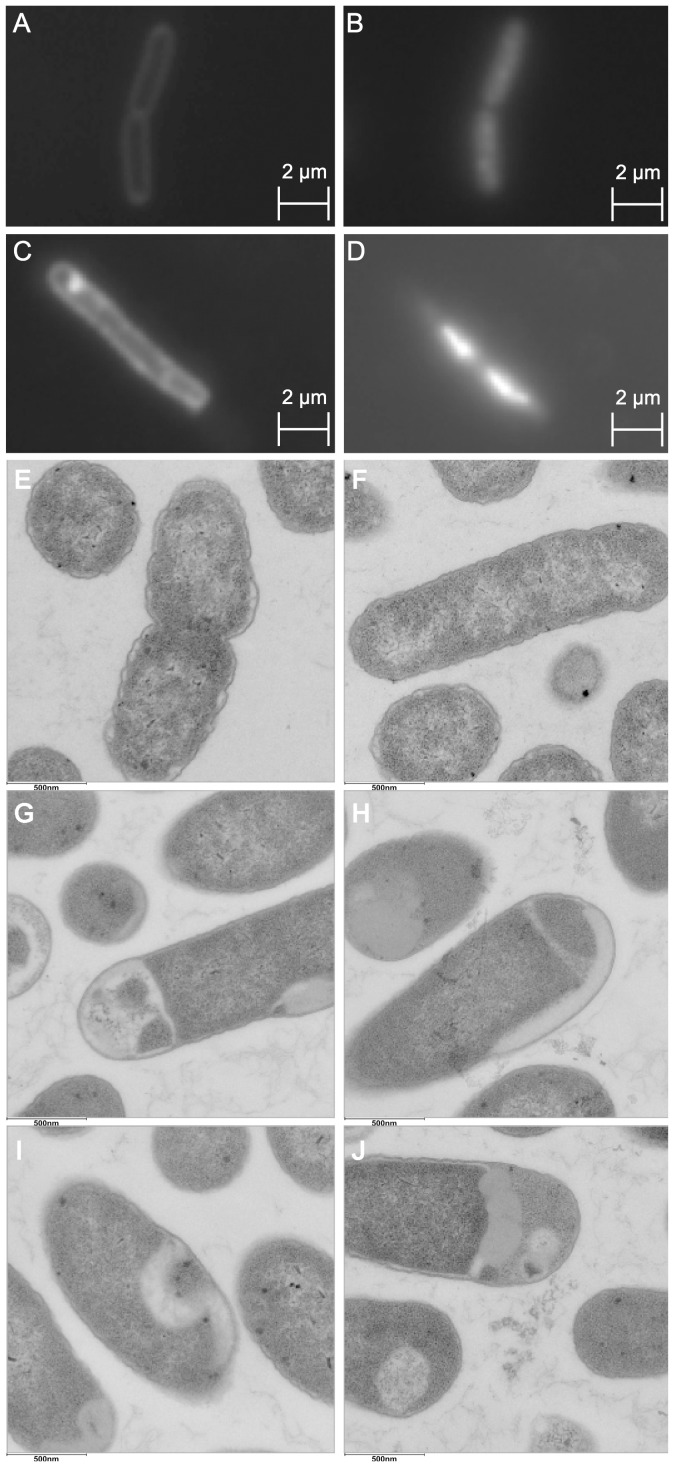
SM10 causes cell filamentation, membrane alteration and DNA condensation. *E. coli* MG1655 was incubated in MHB for 1.5 hr in the presence of 10 µg/ml SM10 (**C**, **D**) or SMs solvent, DMSO (**A**, **B**). The membrane and the DNA were stained with FM4-64 (**A, C**) and DAPI (**B, D**). TEM of *E. coli* MG1655 incubated in MHB for 1.5 hr in the presence of DMSO (**E, F**) or 20 µg/ml SM10 (**G–J**). The samples were fixed in 2% glutaraldehyde and then with 1% osmium tetroxide. Samples were dehydrated with alcohol, embedded in epon and sliced. Staining was performed with uranyl acetate and lead citrate. The slices were viewed with a FEI TECNAI 12 TEM.

To further investigate how SM10 kills bacteria, we stained *E. coli* MG1655 SM10-treated cells with DAPI and the lipophilic membrane dye FM4-64. Cells were grown in the presence of SM10 or DMSO (SM10 solvent) for 90 minutes and were observed by epifluorescence microscopy. SM10 induces both DNA condensation and cell filamentation (Figure 2). In addition, SM10 induces membrane alterations, observed as the accumulation of FM4-64 dye in different areas of the bacterial membrane, including apparently intra-cellular staining (Figure 2C).

In order to achieve higher resolution and magnification of cells and their membranes, we visualized SM10-treated bacteria by transmission electron microscopy (TEM). TEM analysis revealed membrane accumulation inside the cell, blebbing, vesicle–like structures, and condensed DNA (Figure 2 G–J and data not shown). In addition, the membrane of SM10-treated cells was much smoother (stretched) than that of untreated cells, and the cytosol was less granular than that of untreated or DMSO-treated cells. The phenotypes observed indicated that the antibacterial effect of SM10 could be partly due to perturbation of the cell envelope. Indeed, *E. coli* MG1655 incubated for 1 hr with SM10 was 100× more sensitive to 1% SDS than the DMSO control (Figure S3).

### Cerulenin inhibits membrane alteration but promotes the death caused by SM10

**Figure 3:**
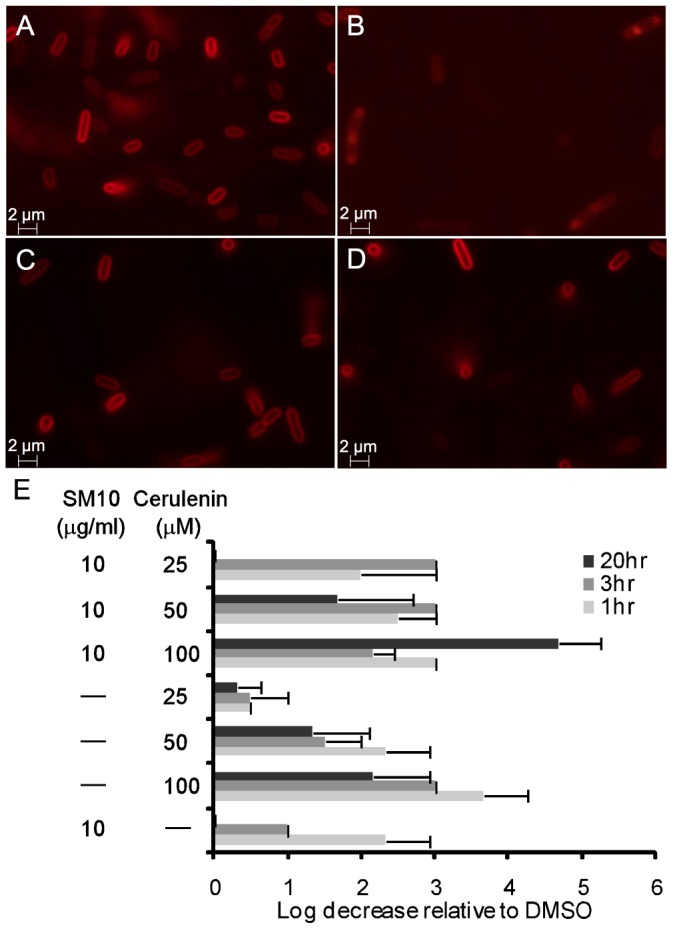
Cerulenin inhibits membrane alteration but does not prevent the death caused by SM10. *E. coli* MG1655 was incubated in MHB for 1.5 hr in the presence of (**A**) DMSO, (**B**) 10 µg/ml SM10, (**C**) 100 µg/ml cerulenin, or (**D**) 10 µg/ml SM10+100 µg/ml cerulenin. The membrane was stained with FM4-64. (**E**) Log sensitivity was calculated relative to DMSO treatment, by testing cell viability after 1 hr, 3 hr, and 20 hr exposure. The absence of a bar at 20 hr reflects cell recovery.

The response to envelope stress often includes induction of biosynthetic enzymes controlling fatty acid, LPS, and phospholipid synthesis [7], [39]. Because the stress induced by SM10 was accompanied by high intensity staining in different membrane areas observed by the lipophillic dye FM4-64, and by membrane accumulation seen by TEM, we tested the effect of inhibiting fatty acid synthesis on these features. Cerulenin, a fatty acid synthesis inhibitor, inhibits 90% of lipid synthesis, 25% of ribonucleic acid and deoxyribonucleic acid synthesis, and has no effect on protein synthesis [40]. The combined treatment of cells with cerulenin and SM10 for 1.5 hours inhibited the membrane blebbing observed by FM4-64 staining (Figure 3). However, cells were hypersensitive to the combined treatment and their viability decreased by 2 logs compared to either single treatment at different concentrations (Figure 3E). These results suggest that fatty acid synthesis is both induced in response to SM10 treatment and is required for bacterial recovery from SM10 treatment. The ^3^H-acetate incorporation results show an increase in phospholipid synthesis after 30 min treatment with SM10, in agreement with the cerulenin results.

### SM10 elicits the envelope stress response

In *E. coli*, three major signaling systems are known to monitor and overcome envelope stress: the transcription factor σE and the two-component signal transduction systems BaeRS and CpxAR [7], [16], [41], [42]. We used strains carrying reporter fusions (*rpoH*::*lacZ*, *spy*::*lacZ*, *cpxP*::*lacZ*) to measure the expression level of genes controlled by these three regulatory pathways following SM10 treatment. Beta-galactosidase activity was monitored following incubation of these strains in MHB for 90 minutes in the presence of SM10. SM10 induced *spy* expression 3-fold, *cpxP* expression 4-fold, and *rpoE* expression by up to 2 fold (Table 1). These results indicated that SM10 induced all of the envelope stress responses. We next tested whether the envelope stress responses are necessary for greater survival after SM10 treatment by determining SM10 sensitivities of strains that are deficient in the *rpoE* or *cpxR* genes compared to the sensitivity of the MC4100 parent strain (because an *E. coli rpoE* null strain is not viable, we used the *ydcQ*-suppressed strain isolated in the Silhavy lab [43]). In parallel, we tested the effect of SM10 on the viability of strains defective in *rpoE* or *cpxR*. The strains were incubated with 10 µg/ml SM10 at 30°C (*rpoE*) and 37°C (*cpxR*) in MHB. Aliquots were taken after 3 hr, diluted, and plated on LB agar plates. The sensitivity of the strains was determined by comparing the viability of the culture treated with SM10 to those that had grown in the presence of 0.5% DMSO. The *rpoE* and *cpxR* deficient strains were found to be 1–3 logs more sensitive to SM10 than the isogenic parent MC4100, respectively (Figure S4, top panel). Thus, SM10 induces envelope stress and the envelope stress response is required, in turn, for greater bacterial survival after SM10 treatment.

**Table 1:** Effect of sublethal concentrations of SM10 on expression of 3 envelope stress responses (β–galactosidase activity expressed in Miller Units, ± SE).

Reporter strain	DMSO (0.5%)	SM10 (10 µg/ml)
***rpoH::lacZ***	770±52	1439±63
***cpxP::lacZ***	3918±89	16018±875
***spy::lacZ***	373±11	1284±17

### SM10 does not cause β-galactosidase leakage but does depolarize the membrane

**Figure 4:**
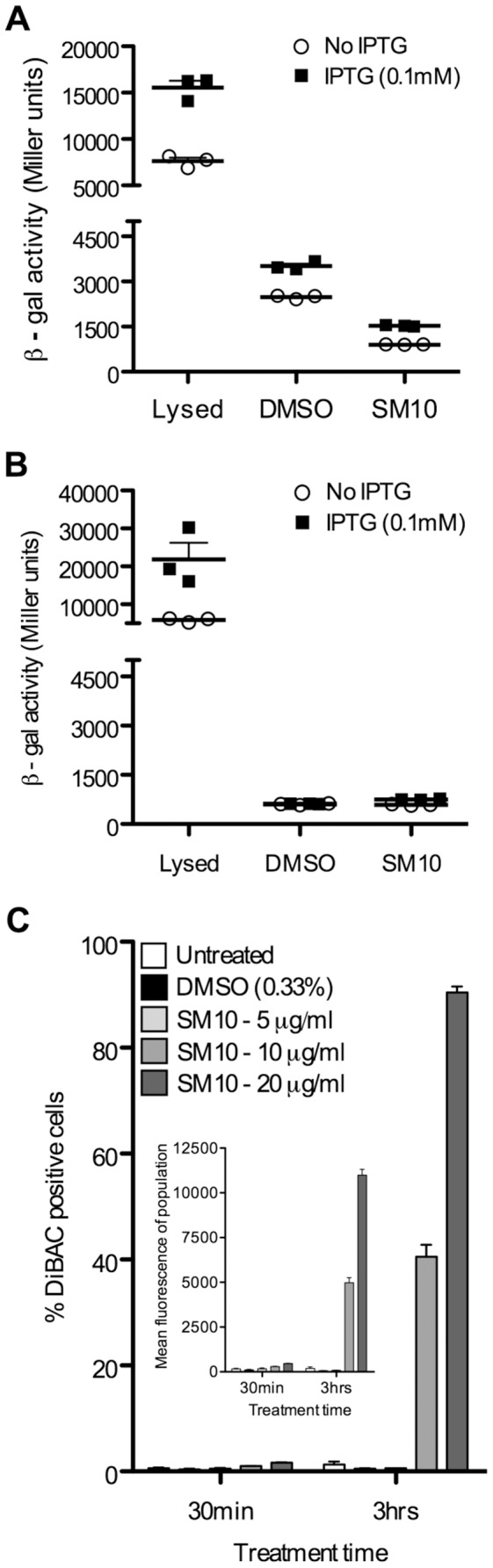
SM10 did not permit leakage of ONPG or β-galactosidase. Log phase *E. coli* MG1655 cells were incubated in the presence of SM10 (10 µg/ml) or DMSO (1%). β-galactosidase activity was induced with IPTG and quantitated after 1 hour, using the substrate ONPG. β-galactosidase activity is expressed in Miller units. “Lysed” indicates cells that were permeabilized using a combination of 1 part 0.1% SDS and 2 parts CHCl_3_ as a positive control. **A.** ONPG to cross the cell membrane freely in cells that were not permeabilized with SDS and CHCl_3_. Cells were induced or not with IPTG, and ONPG was added to the lysed supernatant or to cells treated with DMSO or SM10 (amount). **B.** β-galactosidase did not leak out of SM10-treated cells. The supernatant of cells induced or not with IPTG was assayed for β-galactosidase activity, to test leakage of the enzyme out of the cytoplasm when cells were treated with DMSO or SM10. C. The membrane potential of cells treated with SM10 (or not) was tested using the bis-oxonol indicator molecule DiBAC_4_(3). The fraction of cells showing DiBAC_4_(3) fluorescence and the mean DiBAC fluorescence per cell (inset) are shown; cells were treated with the specified amount of SM10 or with the appropriate amount of DMSO solvent alone.

To further probe SM10's effects on the cell envelope of *E. coli*, we investigated whether SM10 affected permeability. The approach was 2-pronged: to determine if it increased the permeability of *E. coli* to a known non-permeable small molecule roughly the size of 2–3 amino acids, *ortho*-nitrophenyl-β-galactoside (ONPG), and to determine if SM10 treatment caused the leakage of β-galactosidase, a tetramer of 1023 amino acids from cells. To address the first point, we tested whether SM10 could sufficiently permeabilize cells to allow the β-galactosidase substrate ONPG cleavage to reach the cytoplasmic enzyme without the standard CHCl_3_ permeabilization step; β-galactosidase activity did not increase, showing that SM10 treatment did not allow ONPG to enter the cell (Figure 4A). To address the second point, leakage of the LacZ tetramer out of cells, the *lac* operon of MG1655 was first induced with IPTG, then cells were treated with 10 µg/ml SM10 and assayed for β-galactosidase activity after 1.5 hours. Assaying the supernatant showed little enzyme activity, revealing that the induced β-galactosidase was unable to leak out of the cells (Figure 4B).

To test whether SM10 affected caused membrane depolarization, we used the indicator bis-oxonol compound, bis-(1,3-dibutylbarbituric acid) trimethine oxonol (DiBAC_4_(3)). DiBAC enters only depolarized cells and binds to intracellular proteins or membranes. Bound DiBAC exhibits enhanced fluorescence and a red spectral shift (described in more detail in Methods). We added DiBAC to SM10-treated or DMSO treated bacteria and determined the fraction of fluorescing cells (Figure 4C) as well as the mean fluorescence (Figure 4C inset). Both increased with increasing dose of SM10 and increased exposure – only a small fraction of cells were DiBAC^+^ at 30 min, while a much greater fraction of cells were DiBAC^+^ at 10 or 20 µg/ml SM10 (Fig. 4C). The DiBAC assay thus indicates that SM10 depolarizes the *E. coli* membrane, an effect that would be expected to contribute to its inhibition of bacterial growth.

### SM10 induces the SOS response and DNA breaks

**Figure 5:**
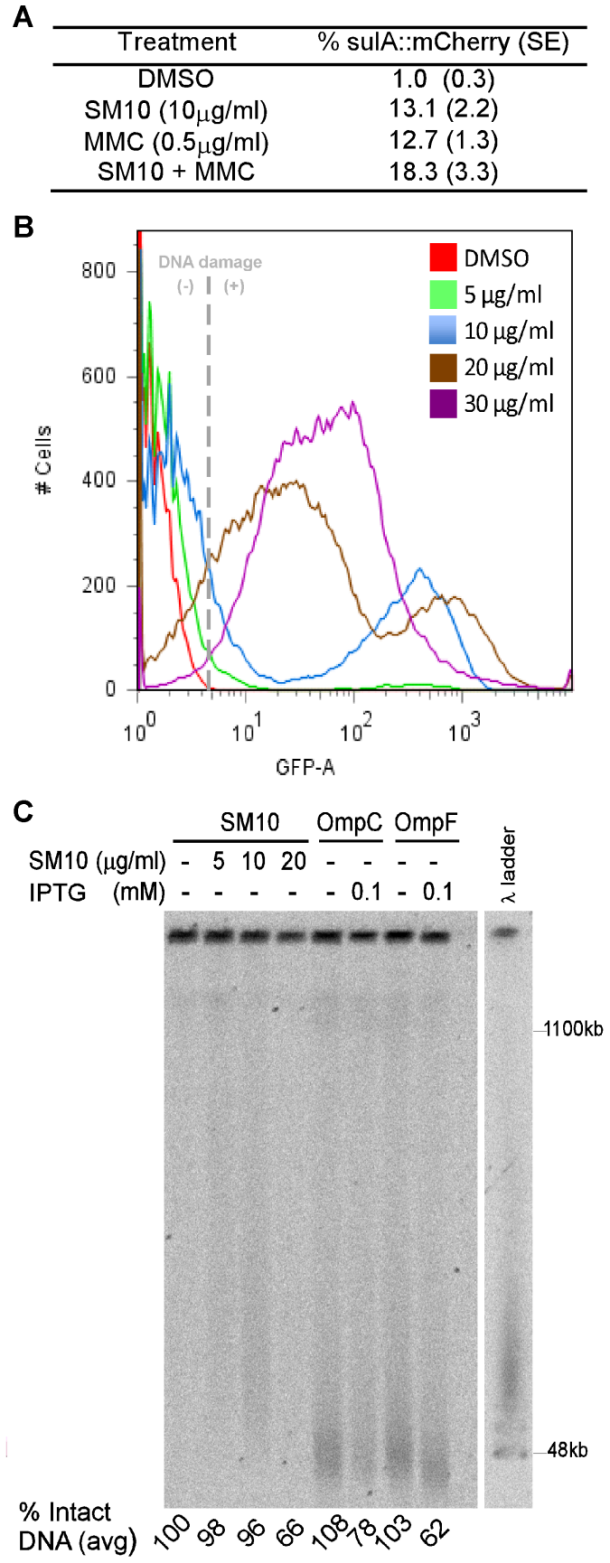
SM10 induces DNA damage in MG1655. For experiments in all panels, bacterial cells were treated with SM10 for 3 hr before the appropriate analysis. **A.** Expression of *sulA*p::mCherry. The *sulA*p::mCherry strain was incubated in MHB in the presence of DMSO, SM10, or MMC. The % of cells positive for mCherry fluorescence cells was determined by flow cytometry. **B.** Measuring DNA breaks induced by SM10 compared to DMSO, using the TUNEL assay. A representative histogram of MG1655 treated bacteria. **C.** DNA damage induced by SM10 treatment or overexpression of OmpC or OmpF includes double strand breaks, as shown by PFGE. Expression of OmpC or OmpF was induced with 0.1 mM IPTG for 3 hr before cells were embedded in agarose.

Filamentation is observed when the SOS response is induced, due to induction of SulA expression and its inhibition of cell division by interactions with FtsZ. To test whether SM10 induces the SOS response, we used a *sulAp*::mCherry reporter fusion to measure *sulA* gene expression. However, to prevent killing due to inhibition of cell division, we used a strain that carries a SulA-insensitive allele of FtsZ, *sulB103* [44]. Fresh subcultures of SS2528 (*sulA*p::mCherry, *sulB103*) were incubated in the presence of SM10 in MHB, and aliquots were taken after 4 hours and analyzed by flow cytometry. As a positive control we used mitomycin C (MMC), a DNA damaging agent that intercalates into DNA and forms adducts and interstrand crosslinks, which trigger the SOS response and DNA repair. SM10 treated cells had a 13-fold increase in SOS response and a similar SOS induction as MMC, while only 1% of the population of DMSO treated cells were positive for mCherry expression (Figure 5A). SM10 combined with MMC had greater SOS induction than either compound alone. Therefore the induction of *sulA* suggests that SM10 induces an accumulation of DNA damage in *E. coli*.

Based on SM10's ability to prevent resolution of HJ intermediates *in vitro* [37] and the similarity between the activities of SM10 and peptide wrwycr [34], we hypothesized that SM10 would also lead to the accumulation of DNA breaks in *E. coli*. To measure DNA damage inside the cell directly, we used the TUNEL assay, which labels free 3′-hydroxyl DNA ends with fluorescein-conjugated dUTP, to measure nicks and double strand breaks inside cells. The percentage of TUNEL-positive cells increased and correlated with increasing SM10 dose and susceptibility to SM10 (Table 2, Figure 5B). A dose-dependent increase in the mean cell size of the population was also observed, correlating with the filamentation caused by SM10 treatment (Figure 5B, Figure S5). At the highest concentrations of SM10, the intensity of the fluorescence decreases although the number of fluorescently-labeled cells decreases. This parallels the results of the pulsed field gels, described below: at high doses of SM10 we observed less total DNA in the lanes as well as in the wells, suggesting degradation of the fragmented DNA. The hypersensitive *cpxR1* mutant and the *rpoE*-deficient mutant accumulated higher levels of DNA damage compared to their isogenic parent MC4100 (Figure S4, bottom panels).

**Table 2:** SM10 treatment increases the fraction of cells with DNA breaks and the amount of DNA breaks per cells, as measured by TUNEL assay.

SM10 dose	Mean FSC^a^ fold difference*	% TUNEL positive	TUNEL fold difference*
**0 (DMSO only)**	844±1	0.35±0.08	1
**5 µg/ml**	2806±3.3	10.9±2.7	31
**10 µg/ml**	3505±4.2	41±3.1	117
**20 µg/ml**	18019±21	62±3.6	177
**30 µg/ml**	32396±38	90±3.9	257

* Relative to DMSO treatment.

Data are the averages of at least 3 independent cultures (± SE).

We also performed pulse field gel electrophoresis (PFGE) to determine whether the DNA damage seen by the TUNEL experiments included double strand DNA breaks (DSB). SM10-treated cells (with 5 or 10 µg/ml) had greater smearing than the DMSO-treated cells, indicating that SM10 causes DSB (Figure 5C). SM10 at 20 µg/ml led to a 34% drop in total DNA relative to DMSO (note that there is less DNA staining in the well of lane 4 compared to the well of lane 1; Figure 5C). This is consistent with DNA degradation after formation of DSB: at lower concentrations of SM10 we observed fragmentation of DNA as evidenced by the smears in lane 2 and 3 whereas the smear disappears at higher concentration shown in lane 4 (Figure 5C). The correlation of viability with the level of DNA damage suggests that DNA breakage may be the primary cause of cell death. Indeed, a Δ*recA* deficient strain was 10 times more sensitive and accumulated twice as many DNA breaks as the parent strain MG1655 (Figure S6). Interestingly, SM10 induced fewer DNA breaks in a *lexA*(ind^−^) strain, which is unable to induce the SOS response, suggesting that enzymes under LexA control may be involved in creating some of the breaks (Figure S6). Thus, SM10 treatment may induce the accumulation of DNA damage either by stabilizing HJ repair intermediates or by causing membrane alterations that themselves lead to DNA damage, perhaps by disrupting electron transport chain components and/or other redox-active proteins.

### Overexpression of OmpC and OmpF causes DNA damage

**Figure 6:**
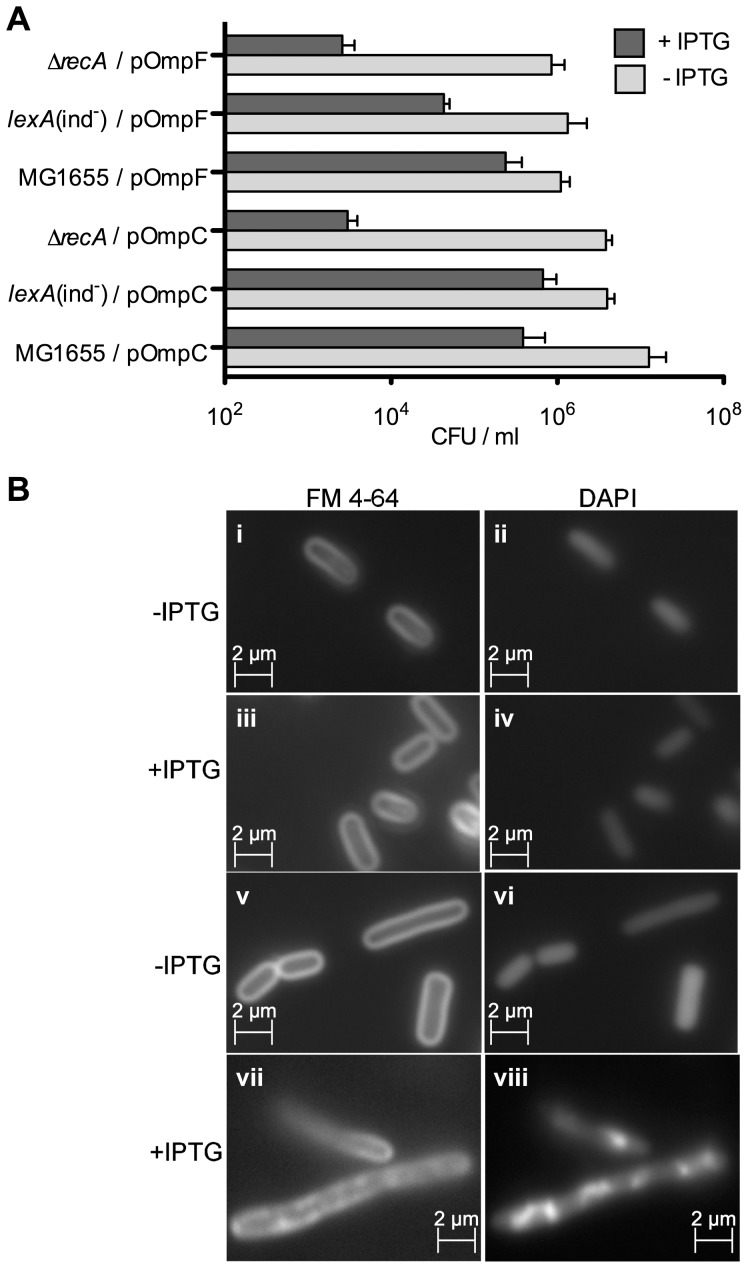
Overexpression of OmpC or OmpF reduces cell viability and perturbs the membrane. **A.** Viability assay of cells overexpressing OmpC or OmpF. Cultures were grown in MHB at 37°C, and overexpression of OmpC or OmpF was induced with 0.1 mM IPTG in log phase and incubated for 3 hr before viable counts were measured. **B.** Overexpression of porins induces membrane alteration. MG1655 cells carrying a high-copy plasmid encoding OmpC (**v, vi, vii, viii**) or PurE (**i, ii, iii, iv**) were incubated 3 hr in the absence (i, ii, v, vi) or presence of 0.1 mM IPTG (iii, iv, vii, viii). The membrane and the DNA were stained with FM4-64 (i, iii, v, vii) and DAPI (ii, iv, vi, viii), respectively.

In order to distinguish between the two alternate potential sources of SM10-induced DNA damage, we tested whether induction of the SOS response and DNA damage can be detected in cells in which envelope stress had been induced in a drug-independent way. Overexpression of porins was the condition in which induction of envelope stress was originally described [17], [45]–[47]. Therefore, DNA damage was analyzed in *E. coli* MG1655 carrying a high copy number plasmid encoding either OmpC or OmpF. The SOS response was tested using a *sulA*p::mCherry strain transformed with either of the porin plasmids. Overexpression of porins was induced by incubation with 0.1 mM IPTG for 3 hours. As a control, we overexpressed two cytosolic proteins, FolD and PurE, transformed into the same genetic background. The *sulA* promoter was induced specifically when cells overexpressed porins, and no change in *sulA* expression was observed in cells overexpressing the cytosolic proteins (Table 3). Even more dramatic was the effect on DNA damage measured by TUNEL assay and PFGE (Table 3, Figure S7). Overexpression of *ompC* or *ompF* resulted in a very large fraction of bacteria suffering DNA damage measured by the TUNEL assay (64±5% and 78±8%, respectively), compared to *purE* or *folD* (1±0.85% and 1.9±0.8%, respectively). PFGE revealed that overexpression of *ompC* or *ompF* lead to DSB, with a loss of intact DNA from the wells of 22% and 38% respectively (Figure 5C). In some cases, we observed DNA damage in cells carrying plasmid-encoded porins even without the addition of IPTG (Table 3, Figure 5C); this is attributable to the leakiness of the p*lac* promoter and the high copy number of the plasmids, which also explains the high basal level of DNA damage observed for pOmpC and pOmpF. The high DNA damage observed coincides with a 10-fold decrease in viability of strains overexpressing OmpC and OmpF (Figure 6A). Similar to SM10-treatment, overexpression of porins in Δ*recA* strain decreased viability by 100 fold (Figure 6A). In addition, strains overexpressing OmpC or OmpF, but not those overexpressing FolD or PurE, exhibited filamentation, DNA condensation (seen after DAPI staining) and accumulation of FM4-64 dye in different areas of the bacterial membrane (Figure 6B), and a larger fraction of anucleate cells (data not shown). These results demonstrated that membrane cues themselves can induce DNA damage, and suggested that the effect of SM10 on DNA damage levels could be a response to membrane alteration rather than a consequence of interfering with DNA repair.

**Table 3:** Overexpression of OmpC or OmpF, but not FolD or PurE, induces the SOS response (mCherry^+^) and increases the fraction of cells with DNA breaks (TUNEL^+^).

Gene overexpressed	Fold difference of mCherry^+^ cells*	% TUNEL^+^ cells	
		− IPTG	+ IPTG
***purE***	1.0±0.05	0.7±0.35	1±0.85
***folD***	1.0±0.11	0.2±0.03	1.9±0.8
***ompC***	7.6±1.41	17±7	64±5
***ompF***	9.9±2.15	17±12	78±8
***none+MMC*** *	31	nd	nd

* Relative to signal of cells grown with no IPTG; as a positive control, cells were treated with 1 µg/ml mitomycin C (MMC).

The data is from 3 experiments comprising 3 independent measurements per day (± SE).

### Membrane stress induces the production of reactive oxygen species

**Figure 7:**
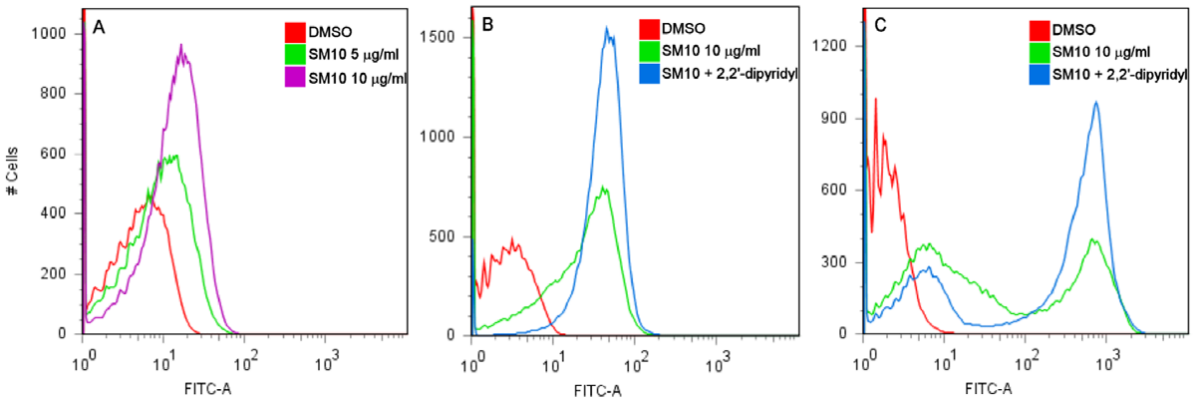
SM10 induces the production of hydroxyl radicals. *E. coli* MG1655 was incubated in MHB at 37°C for 1.5 hr in the presence of SM10 (**A**) alone or in combination with the iron chelator dipyridyl. The cells were washed and incubated with HPF, for quantification of hydroxyl radicals (**A, B**) or processed to quantify DNA breaks (**C**) with the TUNEL assay.

Since SM10 triggers both membrane alteration and DNA breaks, we tested whether reactive oxygen species (ROS), particularly hydroxyl radical production, plays a role in SM10-induced damage. ROS can damage both DNA and membranes [1]. Kohanski and colleagues [26] have suggested that hydroxyl radical generation contributes to the mechanism of killing of bactericidal antibiotics. This was proposed as a common mechanism for all bactericidal drugs, whereby antibiotics cause a surge in the TCA cycle, leading to increased superoxide production [26]. This in turn would damage iron-sulfur clusters, causing an increase in internal iron and Fenton reaction-mediated hydroxyl radical formation [26]. We examined if the toxicity of SM10 was accompanied by hydroxyl radical production. Early exponential phase MG1655 cultures were incubated with SM10 and stained with hydroxyphenyl fluorescein (HPF). HPF was reported to be oxidized by hydroxyl radicals with high specificity, generating free fluorescein [48]. Fluorescence in individual cells was analyzed by flow cytometry. Incubation of *E. coli* with SM10 at a concentration of 5 µg/ml and 10 µg/ml (sublethal concentrations) for 1.5 hours resulted in 6.7±2.3% and 16.1±1.8% cells positive for hydroxyl radical production, respectively (Figure 7A). Interestingly, attempting to inhibit the Fenton reaction and hydroxyl radical formation with the iron chelator 2,2′-dipyridyl (500 µM) resulted in even higher hydroxyl radical formation (Table 4, Figure 7B) and higher levels of DNA damage (Figure 7C). This coincides with a 1–2 log decrease in viability after co-treatment with SM10 and dipyridyl compared to SM10 only (Table 5).

**Table 4:** Effect of dipyridyl on the fraction of HPF^+^ cells in conjunction with SM10 treatment or overexpression of OmpC.

% (+) HPF		Dipyridyl concentration (µM)	
Treatment	0	250	500
**MG1655+DMSO**	0.69±0.16	0.06±0.06	0.2±0.04
**MG1655+SM10**	23±3.8	38±2.5	53±9.6
**OmpC**	2.9±0.66	2.15±1.0	0.53±0.12
**OmpC+0.1 mM IPTG**	25±2.5	17.2±2.5	14.2±3.3

Data are the average of at least 3 independent cultures (± SE).

**Table 5:** Effects of dipyridyl on viability of cells incubated for 3 hr with SM10 or cells over–expressing OmpC, given as the log decrease in cfu/ml.

Dipyridyl	MG1655		OmpC		PurE
(µM)	DMSO	SM10	(−) IPTG	(+) IPTG	(+) IPTG
**0**	-----*	1±0.0	0±0.0	2±0.0	-----*
**250**	0±0.0	2±0.0	0±0.0	1.17±0.17	0±0.0
**500**	0±0.0	2.67±0.33	0±0.0	1.17±0.17	0±0.0

* Decrease in viability relative to DMSO treatment for SM10, or to cells overexpressing the cytoplasmic protein PurE (over–expressing control strain for OmpC).

Data are the average of at least 3 independent cultures (± SE).

Hydroxyl radical formation was also induced by overexpression of porins in the absence of SM10 treatment (Table 6). Early exponential-phase cultures of MG1655 carrying high copy number plasmids encoding the porins OmpC or OmpF, or PurE or FolD as negative controls, were incubated with 0.1 mM IPTG for 3 hr and analyzed using the HPF assay. While the magnitude of the positive signal was relatively low and did not change upon addition of IPTG in the control strains, the background level of HPF^+^ cells in the porin-overexpressing strains was higher and increased 8-fold with the addition of IPTG in the strains overexpressing OmpC or OmpF. Interestingly, in contrast to the effect of dipyridyl on SM10 treated cells, addition of dipyridyl reduced the hydroxyl radicals in cells overexpressing OmpC and the viability of the cells increased accordingly (Tables 4, 5). This suggests that, although both SM10 and overexpression of porins induce envelope stress, DNA damage, hydroxyl radical formation and/or ROS, and eventual death, the involvement of iron and the mechanism that leads to the production of reactive oxygen species may differ between SM10 treatment and porin overexpression.

**Table 6:** Overexpression of OmpF and OmpC induces hydroxyl radical formation.

	Aerobic Growth		Anaerobic Growth	
Gene overexpressed	(−) IPTG	(+) IPTG	(−) IPTG	(+) IPTG
***folD***	1.5±0.6	1.3±0.5	ND	ND
***purE***	1.84±1.1	1.2±0.22	1.6±0.4	0.4±0.1
***ompC***	2.9±0.7	25±2.5	0.5±0.2	1.3±0.4
***ompF***	5.8±1.4	29±7.2	ND	ND

Data are the average of at least 3 independent cultures (± SE).

The Collins group observed that aminoglycoside treatment affected genes regulated by the ArcAB two-component signal transduction system as well as genes associated with the response to mistranslation of proteins, an effect of aminoglycosides [26]. They reported that *cpxA* and *cpxR* mutants did not generate a positive HPF or DiBAC signal, and that *degP* and *arcA* mutants showed decreased HPF and DiBAC signals. They interpreted these and other results as evidence that CpxA may phosphorylate ArcA, and that activation of ArcA in turn led to activation of respiratory systems and a physiological state resulting in the formation of hydroxyl radicals and ultimately cellular death [26]. We therefore tested the effect of SM10 treatment in an *arcA* mutant strain expecting, based on the Collins work, to see amelioration of DNA damage. Instead, the *arcA* mutant showed a higher fraction of TUNEL^+^ cells than the isogenic *arcA* wild type strain (Table S1).

### SM10 treatment and porin overexpression cause DNA damage even in anaerobically-grown cells

**Figure 8:**
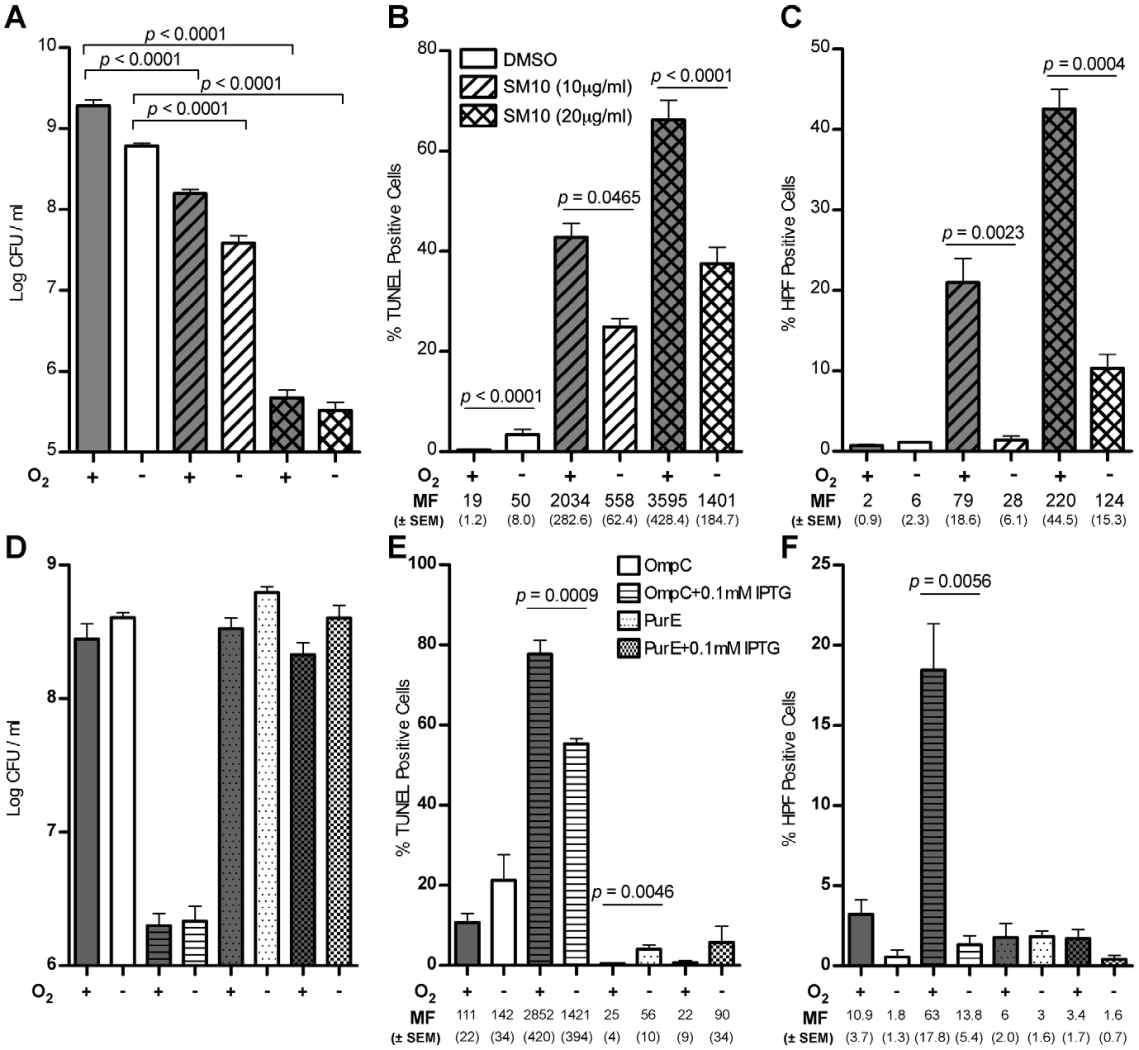
Comparing the effect of SM10 treatment or OmpC overexpression in aerobically- and anaerobically- growing *E. coli*. MG1655 cells were grown for 3 hr at 37°C, with shaking. Anaerobic cultures were grown using an anaerobic hood and/or in a closed-vial-system, in both cases after sparging the O_2_ gas with argon. The legend for panels A, B and C is shown in panel B. The legend for panels D, E and F is shown in panel E; in addition, the presence of O_2_ is indicated both under the X axes as well as by the darker shading of the bars denoting aerobically-grown cells. (**A**) Lethality of SM10 treatments was not greatly affected by lack of oxygen. (**B**) SM10 caused about 40% fewer DNA breaks in MG1655 cells grown anaerobically, as quantified by TUNEL assay. (**C**) SM10-induced formation of ROS in MG1655 cells, visualized by the HPF assay, is negligible in anaerobically–grown MG1655 cells. (**D**) Overexpression of OmpC but not of PurE causes lethality in either aerobically- or anaerobically-growing cells. (**E**) Over-expression of OmpC anaerobically results in about half the DNA breaks observed compared to aerobically, as quantified by TUNEL assay. (**F**) Reactive oxygen species are produced in aerobically-growing cells over-expressing OmpC, but not PurE.

If all of the DNA damage related to envelope stress was due to generation of ROS, both loss of viability and DNA damage should only occur aerobically. To further investigate the role of oxygen in the killing mechanism of SM10, cells were treated under anaerobic conditions to permit comparison with aerobically-grown cells. Anaerobic growth was achieved in an anaerobic hood and/or in an air-tight vial system, in both cases after sparging the O_2_ gas. All overnight cultures were grown anaerobically using the vial-system. The overnight vial cultures were moved into the anaerobic hood, where the subculture was made; anaerobic experiments were performed in the same manner as their aerobic counterparts. The lethality of SM10 treatment was not abated by anaerobic growth (Figure 8A and 8D). While the absence of O_2_ led to a statistically significant decrease in the fraction of cells with DNA breaks relative to aerobically grown MG1655, measured by TUNEL assay, at least 35% of cells suffered DNA breaks anaerobically as well (Figure 8B). At a SM10 concentration of 20 µg/ml, there was only a 33% decrease in the number of cells that were TUNEL-positive (Figure 8B). Measuring the SOS response using the *sulA*p::mCherry strain showed that ∼30% of anaerobically-grown SM10-treated cells induced the SOS response compared to aerobically-grown cells. By comparison, 50% of anaerobically-grown cells treated with a drug known to damage DNA both aerobically and anaerobically, norfloxacin, induced the SOS response compared to aerobically-grown cells (Table 7). As expected, the formation of ROS using the HPF assay revealed a 75% drop or greater in HPF^+^ cells among those grown anaerobically compared to those grown aerobically (Figure 8C). By comparison, inducing endogenous envelope stress anaerobically by over-expression of OmpC led to the same drop in viability (∼2 logs) as in aerobic conditions (Figure 8D). Similar to SM10 treatment, a significant fraction of the population grown anaerobically had DNA breaks (Figure 8E). Anaerobic conditions suppressed the HPF signal induced by over-expression of OmpC in an aerobic environment (Figure 8F).

**Table 7:** SOS induction measured using the *sulA*::mCherry reporter in MG1655 treated with SM10 or norfloxacin aerobically and anaerobically.

Treatment	% *sulA*p::mCherry^+^	
	+ O_2_	− O_2_
**DMSO**	0.8±0.15	0.4±0.03
**SM10 (10 µg/ml)**	8.7±0.33	2.6±0.15
**NFX (0.25 µg/ml)**	83.5±1.5	41.5±1.4

Data are the average of at least 3 independent cultures (± SE).

### Other envelope stress-inducing conditions cause DNA damage

**Figure 9:**
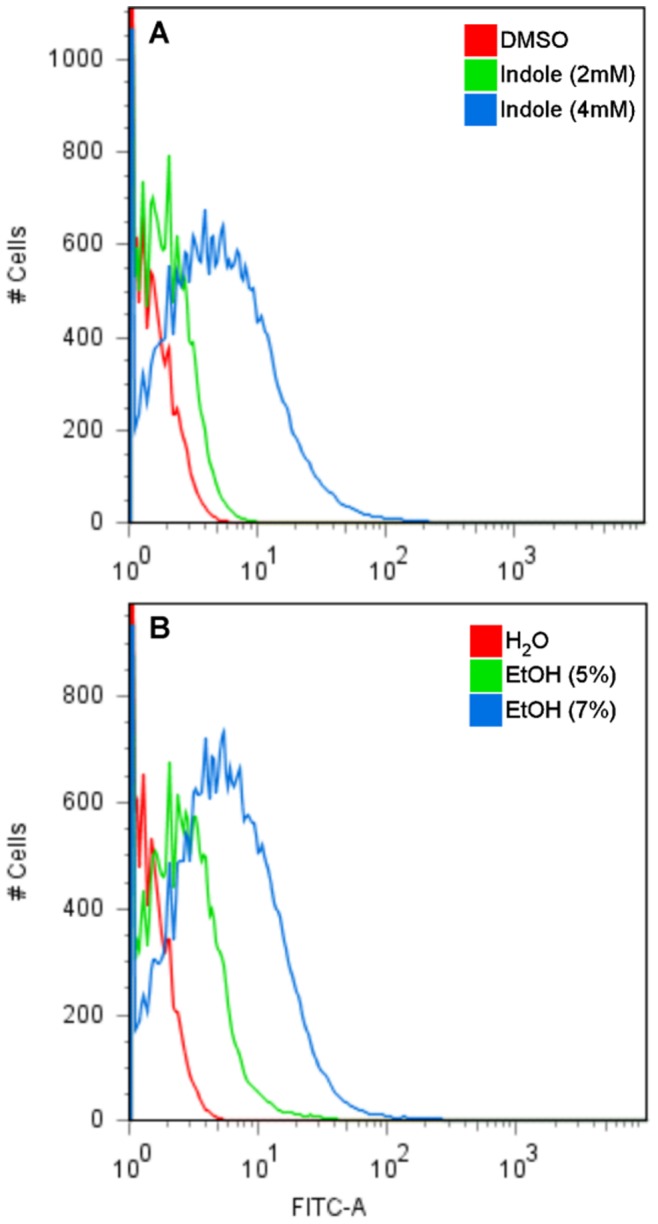
Treatment with indole and ethanol induce DNA damage. MG1655 cells were incubated in MHB at 37°C in the presence of the indicated concentration of indole (**A**) or ethanol (**B**) for 3 hr. The DNA damage was detected using the TUNEL assay.

In order to verify whether DNA damage occurs uniquely during overexpression of porins or whether it is a general effect induced by envelope stress, we tested other known membrane perturbation inducers namely ethanol, indole and elevated pH. Ethanol is known to denature proteins and induce the σ^E^ response [8], [49]. Indole and elevated pH have been shown to upregulate Spy protein levels via induction of the BaeRS system [50], [51] and the Cpx pathway [12], respectively. MG1655 cells grown in the presence of different concentrations of ethanol or indole induced DNA damage and increased cell size (measured by forward scatter) in a concentration-dependent manner (Figure 9). A certain threshold level of stress or damage was needed in order to induce DNA breaks. A low concentration of ethanol (5%) or indole (2 mM) induced a low level of DNA damage (2.7±0.3% and 0.5±0.1%, respectively). Increasing the concentration to 7% ethanol or 4 mM indole resulted in a 5.5× and 48× increase in the number of cells with DNA breaks, respectively, a disproportionate increase with respect to the amount of added stressor. Incubation of MG1655 in an alkaline environment (pH 8.4) also caused a very mild (∼2-fold) increase in the amount of DNA damage (data not shown).

Heat is another condition that can induce membrane stress. RpoE – null strains in which suppressors of lethality arise nevertheless show envelope defects such as hypersensitivity to heat [45]. We tested whether the sensitivity of the *ydcQ*-suppressed *rpoE* mutant strain [43] was accompanied by accumulation of DNA breaks. The cultures were incubated for 3 hours at 3 different temperatures (30°C, 37°C and 45°C) and the fraction of cells sustaining DNA damage was analyzed using the TUNEL assay. The mean fluorescence values are presented in Table 8. While the DNA damage in the control strain MC4100 remained relatively low and similar at all temperatures tested, the *rpoE*-null strain had a high fluorescence value (>13× greater than the isogenic MC4100) at 45°C.

**Table 8:** DNA breaks present in Δ*rpoE* versus its isogenic parent strain, MC4100, grown at different temperatures, determined using TUNEL.

Temp	30°C	37°C	45°C
**MC4100**	21±1.7	24±0.6	29±0.9
**Δ** ***rpoE ydcQ*** *	23±0.5	25±1.2	390±21.4

Values represent mean fluorescence of the population.

* In *E. coli*, *rpoE* is essential but the lethality of the deletion can be suppressed by the *ydcQ* mutation [43].

Data are the average of at least 3 independent cultures (± SE).

## Discussion

While investigating the mode of action of a novel antibiotic molecule, we discovered that treatments that induce envelope stress, including endogenously-induced envelope stress caused by over–expression of outer membrane proteins, induce DNA damage. Exogenous treatments that similarly induce both envelope stress and DNA damage include indole, heat, and low concentrations of EtOH, as well as our small molecule SM10 (TPI1609-10).

The SM10 molecule was identified from mixtures of small molecules based on its activity in inhibiting site-specific recombination mediated by the phage lambda Int protein by trapping Holliday junctions, similarly to the previously characterized peptide WRWYCR and its d-amino acid isomer wrwycr [37]. Bulk DNA synthesis was not affected by SM10 treatment, while bulk RNA and protein synthesis were only slightly inhibited by SM10 at 20 µg/ml (Figure S2B, D). In contrast, incorporation of ^14^C-labeled acetate into phospholipids was somewhat increased by treatment with SM10 (Figure S2C). at 5 and 10 µg/ml concentrations of SM10 respectively after 30 min of incorporation. Cells treated with SM10 accumulate intracellular membrane structures and “pockets” that are visible both by FM4-64 staining and TEM. The envelope damage correlates with loss of proton motive force, and new synthesis of fatty acids appears to be required for cell survival in the presence of SM10, since cerulenin, which inhibits fatty acid synthesis [40], is synergistically lethal with SM10 treatment

We show here that while SM10, like wrwycr [34], also causes DNA damage, it also causes envelope stress and somehow generates reactive oxygen species. Thus we are faced with a dilemma of causality – is all the DNA damage we observe related to envelope stress alone, or is some of the DNA damage indeed due to interfering with DNA repair. Links between envelope stress and DNA damage have been suggested previously, for example by data showing that bile salts, known to perturb the membrane and thus inducing the ESR, also activated the SOS response [52]. In *Vibrio cholerae*, cell perturbations that led to ESR activation also induced oxidative stress [53], which is known to cause DNA damage. The Cohen lab showed that ampicillin treatment induced the SOS response [22], and Kohanski and colleagues showed that treatment with penicillin and aminoglycoside antibiotics activated the SOS response [26], [27]. However, all these cases involved treatment with exogenous agents with antibiotic activity. To our knowledge, we are the first to show that endogenously induced envelope stress caused by over-expression of outer-membrane proteins also induced the SOS response and accumulation of DNA breaks. This finding has broad implications for the consequences of environmental conditions on the survival of bacteria, and the observations of mutagenesis accompanying starvation, stationary phase, and growth in the presence of the mammalian immune system may be causally interrelated to direct damage of DNA in conditions that affect the cell envelope. Exactly how membrane damage causes DNA damage must be the topic of future studies.

One possible explanation of how envelope stress may induce DNA damage was suggested by the Collins lab, namely that aminoglycosides lead to inhibition of translation and accumulation of misfolded proteins in the inner membrane, leading to damage of components of the electron transport chain and consequently to an increase in the formation of ROS [26]. Kohanski and collaborators have also proposed a model connecting ESR to ROS formation by cross-talk between CpxA and ArcA, leading to ArcA activation and subsequently provoking an increasingly oxidative stress state within the cell [27]. We disfavor this latter model because even nearly identical two-component signaling systems are unlikely to cross-talk [54], [55]. Moreover, unlike in the case of aminoglycosides [27], the *arcA* mutant suffers greater, not less, SM10-dependent DNA damage (Table S1).

The most deleterious ROS, hydroxyl radicals, target DNA due to their formation by iron-dependent Fenton chemistry and the association of iron with DNA. Indeed, our data is consistent with Fenton-chemistry generation of ROS, since essentially all of the HPF oxidation disappears when anaerobically-growing cells were treated with SM10 or overexpressed OmpC. However, a significant fraction of cells grown anaerobically still suffer DNA damage, despite the lack of oxygen. Moreover, neither the lethality of SM10 nor of OmpC over-expression is abated significantly anaerobically. Thus, while loss of viability may be due to a combination of factors, DNA damage appears to be a critical component both aerobically and anaerobically, and any explanation of the lethal effect of inducing envelope stress cannot depend solely on the presence of reactive oxygen and has to account for the anaerobic toxicity.

We propose a model that accounts for both the anaerobic effects of SM10 treatment and OmpC protein overexpression and the DNA damage associated with wrwycr and SM10 as well as overexpression of OmpC. We have shown that wrwycr causes accumulation of intracellular iron, transient loss of membrane potential, and leakage of potassium from cells ([56]; Naili, Rostron and Segall, ms. submitted). Like wrwycr, SM10 also causes loss of membrane potential (Figure 4C). Preliminary data showed that SM10 also causes potassium leakage out of cells: cells treated with 40 µg/ml SM10 for 20 minutes led to a 4.9 fold drop in intracellular K^+^, compared to 8.5 fold drop in cells treated with 32 µM wrwycr. Cells compensate for potassium loss by increasing uptake of H^+^, which causes a drop in intracellular pH. Acidifying the cytoplasm increases the rate of depurination of DNA [57]. The resulting abasic sites, if they overwhelm the base excision and nucleotide excision repair systems, will result in single strand DNA breaks, induction of the SOS response, and collapse of replication forks, which transforms the single strand breaks to double strand breaks [2], [58]. If this model is correct we would expect that, like peptide wrwycr, SM10's lethality will be greatly amplified when the treated bacteria are in an acidic environment [60].

Thus, while envelope stress induction and the attendant increase in DNA damage appear to be an important component of the mechanism of action of SM10, envelope stress does not explain all aspects of SM10's mechanism of action. For example, the addition of the iron-chelator dipyridyl, which had the expected protective effect in the case of OmpC and OmpF over-expression, had a negative effect in SM10-treated cells. Perhaps SM10, like peptide wrwycr which it resembles functionally *in vitro*, sequesters iron and makes it bio-unavailable to cells but still able to react with ROS [56]. In the case of peptide wrwycr, we showed that cells react to being starved of iron and derepress the Fur regulon, If this is also the case with SM10, dipyridyl may severely exacerbate the shortage of iron. However, we have no direct evidence of interactions between either wrwycr or SM10 and iron.

The experiments reported here have characterized the effects on *E. coli* of a small molecule, SM10, screened for its ability to inhibit phage λ site-specific recombination by trapping HJ intermediates [37]. Based on our results, we conclude that SM10's bactericidal effect can be attributed to a combination of effects on the membrane and accumulation of DNA damage, perhaps due aerobically to increased levels of ROS. Both aerobically and anaerobically, we propose that the lethality may be due to a decrease in intracellular pH and the resulting accumulation of abasic sites in DNA that overwhelms the cell's DNA repair systems and, concomitantly, accumulates HJ targets for the small molecules.

## Methods

### Strains and bacterial culture methods

Bacterial strains used in this work and their sources are listed in Table 9. Strains were maintained on LB agar plates and cultures were grown, usually aerobically, in Mueller-Hinton broth (MHB; Difco) as specified. Anaerobic cultures were grown overnight in argon-sparged MHB inside a closed-vial system, using a Suba-Seal septa (Sigma) and 2 ml vials. A vacuum was pulled using a 22 gauge needle, for 2 minutes followed by 5 minutes of argon sparging, repeated twice. Anaerobic cultures were subcultured and treated inside an anaerobic chamber (MBRAUN LABstar 1200) or in the closed-vial system. To test our anaerobic conditions, we used the obligate aerobe, *Micrococcus luteus*, as a control – indeed, our conditions did not permit the growth of these bacteria.

**Table 9:** Strain list.

Designation	*E. coli* K12	Genotype	Source
EM497/G652	MG1655	*rph1* λ^−^	S. Maloy
CAG16037/G800	MG1655	MG1655 Δ*(araCOIBA, leu)7696 araD139 galK galU hsdR Δ(lac)X74 recA56 rpsL* Φλ[*rpoHP3′-lacZ+*] ts	C. Gross
MC4100/G832	MC4100	F^−^ *araD139* Δ*(argF-lac)U169 rpsL150 relA1 flbB5301 deoC1 ptsF25 rbsR*	T. Silhavy
TR50/G833	MC4100	attλRS88 Φ[*cpxP′-lacZ^+^*]	T. Silhavy
TR51/G834	MC4100	*cpxR1*::Spc	T. Silhavy
TR531/G835	MC4100	*att*λRS88 Φ[*spy′-lacZ^+^*]	T. Silhavy
NR982/G839	MC4100	*rpoE*::Cam *ydcQ*::Kan	T. Silhavy
SS2528/G775	AB1157	*sulA*p-mcherry *gal-7*6::Tn*10*	S. Sandler
EDT1620	MG1655	pASKAHis*ompC* (cam^R^)	Laboratory collection
EDT1611	MG1655	pASKAHis*ompF* (Cam^R^)	Laboratory collection
EDT1663	MG1655	pASKAHis*purE* (Cam^R^)	Laboratory collection
EDT1662	MG1655	pASKAHis*folD* (Cam^R^)	Laboratory collection
EDT1667	AB1157	*sulA*p-mcherry *gal-76*::Tn*10*/pASKAHis*ompC* (Cam^R^)	Laboratory collection
EDT1668	AB1157	*sulA*p-mcherry g*al-76*::Tn*10*/pASKAHis*ompF* (Cam^R^)	Laboratory collection
EDT1671	AB1157	*sulA*p-mcherry *gal-76*::Tn*10*/pASKAHis*purE* (Cam^R^)	Laboratory collection
EDT1669	AB1157	*sulA*p-mcherry *gal-76*::Tn*10/*pASKAHis*folD* (Cam^R^)	Laboratory collection

When strains were obtained from other labs, the original strain name is given followed by our lab designation.

### Small molecules

The stock solution of SM10 (1 mg/ml concentration) was dissolved in 50% DMSO. Its synthesis and purification are described elsewhere [37]. In the solvent controls for all experiments, we used the DMSO concentration corresponding to the highest small molecule concentration.

### Viability assays

Overnight cultures in MHB were subcultured 1∶100 and incubated 1 hr at 37°C. The cultures were divided into 96 well microtiter plate containing the desired concentration of SM10 in a final volume of 150 µl. The plate was shaken at 37°C and aliquots were taken after 1 hr, 3 hr and 20 hour, diluted in PBS, and spotted on LB agar. Log sensitivity was calculated relative to parallel DMSO-treated cultures included in each experiment.

### Metabolite Incorporation Assays

Overnight cultures were subcultured 1∶500 in MHB or MOPS media supplemented with 0.2% glucose and grown to an OD_535_ of 0.1, when radiolabeled metabolites were added to monitor SM10's effects on replication, transcription, translation, and phospholipid synthesis. The metabolites added per 0.1 ml of culture were: 50 nCi of [methyl-^3^H] thymidine (71.7 Ci/mmol; Perkin Elmer Corp.) to measure incorporation into DNA; 250 nCi of [5,6-^3^H] uridine (35.6 Ci/mmol; Perkin Elmer Corp.) to measure incorporation into RNA; 125 nCi of [4,5-^3^H] leucine (146.5 Ci/mmol; Perkin Elmer Corp.) to measure incorporation into protein; and 10 µCi [^3^H] acetate (150 mCi/mmol; American Radiolabeled Chemicals Inc.) or 1 µCi [^14^C] acetate (106 mCi/mmol American Radiolabeled Chemicals Inc.) to measure incorporation into phospholipids. DMSO, SM10, and the appropriate positive control treatment were immediately added after the radioactive chemicals. Samples (0.1 ml) were taken at specified time intervals. In the case of the replication, transcription and translation experiments, cells were lysed by mixing them with 0.1 ml ice-cold 10% TCA, followed by collection of the precipitates on a nitrocellulose membrane using a 96-well vacuum manifold. After washing the precipitates with 0.2 ml of ice-cold 5% TCA, the membrane was dried and cut into “slices” representing each well; each slices was placed in 5 ml of liquid scintillation fluid and radioactivity counts were determined using a Beckmann LS 6500 scintillation counter [34]. In the case of the phospholipid synthesis, cells were lysed by mixing them with 0.6 ml of CHCl_3_∶MeOH (1∶2). After lysis, 0.2 ml CHCl_3_ and 0.2 ml of H_2_O were added to each sample, which separated the aqueous and organic phases. After discarding the aqueous phase, the organic phase was washed 3 times with 0.6 ml of 2 M KCl and 0.1 M NaOAc [59]. Liquid scintillation fluid (5 ml) was added to the organic phase collected from each sample and radioactivity counts were determined using a Beckmann LS 6500 scintillation counter.

### Lysis assay

Overnight cultures in MHB were subcultured 1∶100 and incubated 1 hr at 37°C. The cultures were divided into 96-well microtiter plate containing the desired concentration of SM in a final volume of 150 µl. SDS was added to a final concentration of 1%, the plate was gently shaken (to avoid bubble formation), and the optical density at 630 nm was determined using a Molecular Devices SpectraMax Plus plate reader.

### β-Galactosidase Assay

Overnight cultures in MHB were subcultured 1∶100 and incubated for ∼1 hr at 37°C. The cultures were divided into a 96 well microtiter plate containing the desired concentration of SM10 in a final volume of 150 µl and incubated for 1.5 hr at 37°C. β –Galactosidase activity was measured after 1.5 hr treatment time, and processed as previously described [61].

### Modification of β-Galactosidase Assay to Monitor Membrane Integrity

Overnight cultures in MHB were subcultured 1∶100 and incubated for ∼1 hr at 37°C. The cultures were divided into a 96 well microtiter plate containing the desired concentration of SM10 in a final volume of 150 µl and incubated for 1.5 hr at 37°C. Cells were then pelleted and processed in 2 ways. To determine if β-galactosidase leaked out of the cells the supernatant was processed using the method described by Miller [61] except that we assayed the supernatant (media) rather than the cells. To determine if the non-permeable substrate ONPG was able to traverse the membrane following SM10-treatment, we processed the cultures as described by Miller [61] except that we did not permeabilize the cells with organic solvents.

### Epifluorescence and Transmission Electron Microscopy

Cultures were grown overnight, diluted 1∶100 and treated with SM for 90 min at 37°C. Cells were pelleted and resuspended in PBS. DAPI and FM4-64 were added to a final concentration of 2.5 µg/ml and 5 µg/ml, respectively. Cells were immobilized onto slides by using 30 µls of 0.1% poly-l-lysine. Epifluorescence images were taken using either a Nikon Microphot Light Microscope equipped with an Olympus Magnafire digital camera, or using a Zeiss AxioObserver Z1 equipped with a Hamamatsu ORCA-ER camera controlled by AxioVision version 4 software.

For TEM, cells were treated as described above. The samples were fixed in 2% gluaraldehyde in PBS, rinsed 3× and then postfixed with 1% osmium tetroxide in PBS. Samples were dehydrated with alcohol, embedded in epon and sliced. The slices were stained with uranyl acetate and lead citrate, and viewed with a FEI TECNAI 12 TEM. Images were recorded on a Teitz 214 bottom-mount digital camera.

### SOS Response Induction

Overnight cultures of *E. coli* containing a *sulA*p::mCherry transcriptional reporter fusion were subcultured 1∶100 in MH broth and incubated 1 hr at 37°C. SM10 or MMC were added to the cultures and aliquots analyzed by flow cytometer after 2 hr and 4 hr. For the detection of the SOS response in OmpF and OmpC strains, overnight cultures were diluted 1∶50 in MH broth and incubated for 1.5 hr before adding 0.1 mM IPTG.

### Flow cytometry analysis

Flow cytometry was performed on BD FACSAria desktop cell sorter (Becton-Dickinson, San Jose, CA) with standard 488 nm and 633 nm lasers. A 70 µm nozzle was used to collect all data. At least 50,000 cells were collected for each sample. The following photomultiplier tube settings were used: 400 V (FSC), 320 V (SSC), 625 V (GFP or FITC) for TUNEL, 650–700 V (GFP or FITC) for hydroxyl radicals detection, and 650 V (PE-Texas Red) for *sulA*p::mcherry analysis. Data acquisition and analysis was performed using FACSDiva software (Becton-Dickinson San Jose, CA). In addition to fluorescence parameters, FSC and SSC parameters were collected in all flow cytometry experiments.

### Direct assay for DNA breaks

During the TUNEL assay, free 3′OH ends in DNA are fluorescently labeled. Cells were grown in the conditions specified. The cultures were pelleted immediately after incubation, fixed with 4% paraformaldehyde, and assayed using the In Situ Cell Death Detection Kit, Fluorescein (Roche, Germany), as described [34]. After treatment, cells were quantified using flow cytometry.

### Detecting formation of intracellular oxygen radicals (hydroxyl radicals)

Hydroxylphenyl fluorescein (HPF) becomes oxidized in the presence of oxygen radicals, releasing the fluorescein and allowing you to measure formation of oxygen radicals. Cells were grown in the condition(s) above. Immediately after incubation the cells were pelleted, resuspended in PBS, split into 2 eppendorf tubes, and 1 µl of HPF (H36004; Invitrogen, Inc.) or DMF (solvent of HPF) was added to the tubes. Cells were incubated for 30 minutes at room temperature in the dark, followed by pelleting cells and resuspending in fresh PBS where by samples were quantified using flow cytometry.

### Detection of membrane depolarization

Bis-(1,3-dibutylbarbituric acid) trimethine oxonol [DiBAC_4_(3); Invitrogen (B438)] enters depolarized cells, where it binds to intracellular proteins or membranes and exhibits enhanced fluorescence and a red spectral shift, emitting fluorescence at 516 nm when excited at 493 nm. Cells were grown in the conditions described above. Immediately after incubation, 50 µl of cells were pelleted, resuspended in 100 µl of PBS+10 µg/ml DiBAC_4_(3), and incubated in the dark at room temperature for 15 min. An additional 200 µl of PBS was added and samples were immediately analyzed using flow cytometry.

### Pulse Field Gel Electrophoresis (PFGE)

PFGE was used to detect dsDNA breaks. Cells were grown in the conditions specified. Agarose plugs (1.5% PFGE Agarose (BIO-RAD)) were made with equal volumes of molten, cooled agarose and suspensions of bacterial cultures whose OD_600_ was adjusted to be equal. Cells were lysed within plugs (1 hr at 65°C) and proteins were inactivated using proteinase K treatment for 24–72 hrs at 42°C. Plugs were then washed and cast in 1% PFGE Agarose (Bio-RAD) made with 0.5× TBE. Gels were electrophoresed in a Bio-RAD Chef Mapper XA Pulse Field Electrophoresis System for 22 hrs at 14°C, 6.0 V/cm, 120° included angle, 50–90 second switch time, with a linear ramp. Gels were stained with SYBR Green (200 ml of 1× solution; Invitrogen, Inc.) for 30 minutes, and scanned on a STORM 860 Molecular Imager. ImageQuant 5.2 software was used to quantify the gels. Values were then normalized to “WT” cells “treated” with equal volumes of the DMSO solvent.

## Supporting Information

Figure S1 **MG1655 cells were incubated in the presence of SM10 in minimal media (NCE, 0.2% glucose, 1 mM MgSO_4_, 12 µM FeCl_3_) for defined times then the cultures were diluted and plated on LB.** Log decrease in viable cells was calculated relative to DMSO (SM10 solvent) treatment for 3 independent cultures. The symbols denote the following treatments: **x**'s, DMSO; **triangles**, 5 µg/ml SM10; **circles**, 10 µg/ml SM10; **squares**, 20 µg/ml SM10; and **diamonds**, 30 µg/ml SM10 final concentration.
(DOCX)

Figure S2 **Effect of SM10 treatment on metabolite incorporation in exponentially growing** ***E.coli*** **MG1655.** **A.** Incorporation of ^3^H-thymidine to monitor DNA synthesis. **B.** Incorporation of ^3^H-uridine to monitor RNA synthesis. **C.** Incorporation of ^14^C-acetate to monitor phospholipid synthesis. **D.** Incorporation of ^3^H-leucine to monitor protein synthesis. For the experiments shown in panels A, B and C, cells were grown in MHB; for the experiment shown in panel D, cells were grown in MOPS medium supplemented with 0.2% glucose. The results are expressed as % incorporation, where measured incorporated cpm in SM10-treated cells were normalized to cpm incorporated in DMSO-treated cells.
(DOC)

Figure S3 **SM10 increased bacterial lysis and reduced viability of MG1655 in the presence of SDS.** All cultures were grown in MHB. (**A**) *E. coli* MG1655 cells were incubated for 1 hr at 37°C in the presence of 10 µg/ml SM10 or DMSO. (**B**) Effect of SM10 on bacterial viability in conjunction with 1% SDS treatment. SDS (1%) was added to MG1655 cultures for 3 hours and the viability of the cultures was tested.
(DOCX)

Figure S4 **CpxR- and RpoE-deficient strains are hypersensitive to SM10.** MC4100, *cpxR1* and an Δ*rpoE* (suppressed by a mutation in *ydcQ*) strains were incubated in MHB at 37°C or 30°C, respectively, in the presence of DMSO or 10 µg/ml SM10. Cell viability relative to DMSO treatment was measured after 3 hours.
(DOCX)

Figure S5 **SM10 treatment caused filamentation, detected using flow cytometry.** *E. coli* MG1655 incubated with the indicated concentrations of SM10 for 3 hours. The differences in the cell size parameter, FSC, are presented from the TUNEL experiment whose results are summarized in Table 3.
(DOCX)

Figure S6 **Results of TUNEL and viability assays performed in parallel on SOS deficient strains treated in exponential phase with DMSO or SM10 for 3 hours.** **A.** TUNEL assay results. The strains specified were treated with DMSO or SM10 and DNA breaks were measured using the TUNEL assay (Methods). **B.** Viability assay results. Cultures were diluted, plated and the log decrease in viable cells calculated relative to DMSO treated cells. Data from 3 independent cultures per strain are shown.
(DOCX)

Figure S7 **Overexpression of OmpC or OmpF causes an increase in free 3′OH DNA ends.** Representative histograms are shown from TUNEL assays of MG1655 carrying high copy number plasmids encoding porins (OmpC or OmpF) or cytosolic proteins (PurE or FolD). Cells were incubated for 3 hr in the absence or in the presence of 0.1 mM IPTG. The TUNEL assay results were quantified using flow cytometry.
(DOCX)

Table S1 **TUNEL assay of SM10-treated Δ** ***arcA*** **cells or wild type isogenic cells.**
(DOCX)
