# Supplementary figures and images for: Similarities between Exogenously- and Endogenously-Induced Envelope Stress: The Effects of a New Antibacterial Molecule, TPI1609-10
Source: PLoS One. 2012 Oct 11;7(10):e44896. doi: 10.1371/journal.pone.0044896 (PMC3469575; doi:10.1371/journal.pone.0044896)

**
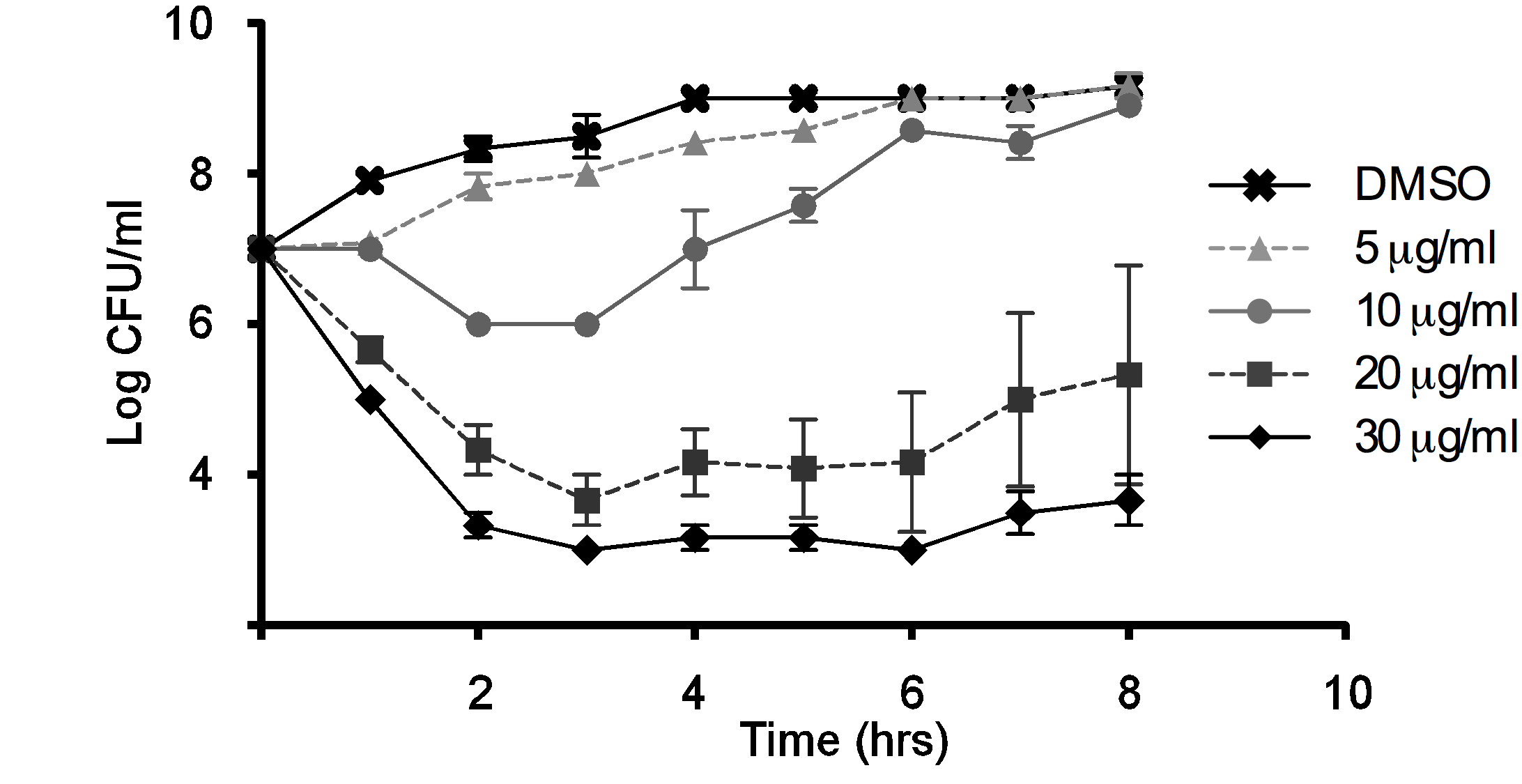
**

Supplement: Figure S1 — MG1655 cells were incubated in the presence of SM10 in minimal media (NCE, 0.2% glucose, 1 mM MgSO4, 12 µM FeCl3) for defined times then the cultures were diluted and plated on LB. Log decrease in viable cells was calculated relative to DMSO (SM10 solvent) treatment for 3 independent cultures. The symbols denote the following treatments: x's, DMSO; triangles, 5 µg/ml SM10; circles, 10 µg/ml SM10; squares, 20 µg/ml SM10; and diamonds, 30 µg/ml SM10 final concentration. (DOCX) [file pone.0044896.s001.docx]

**
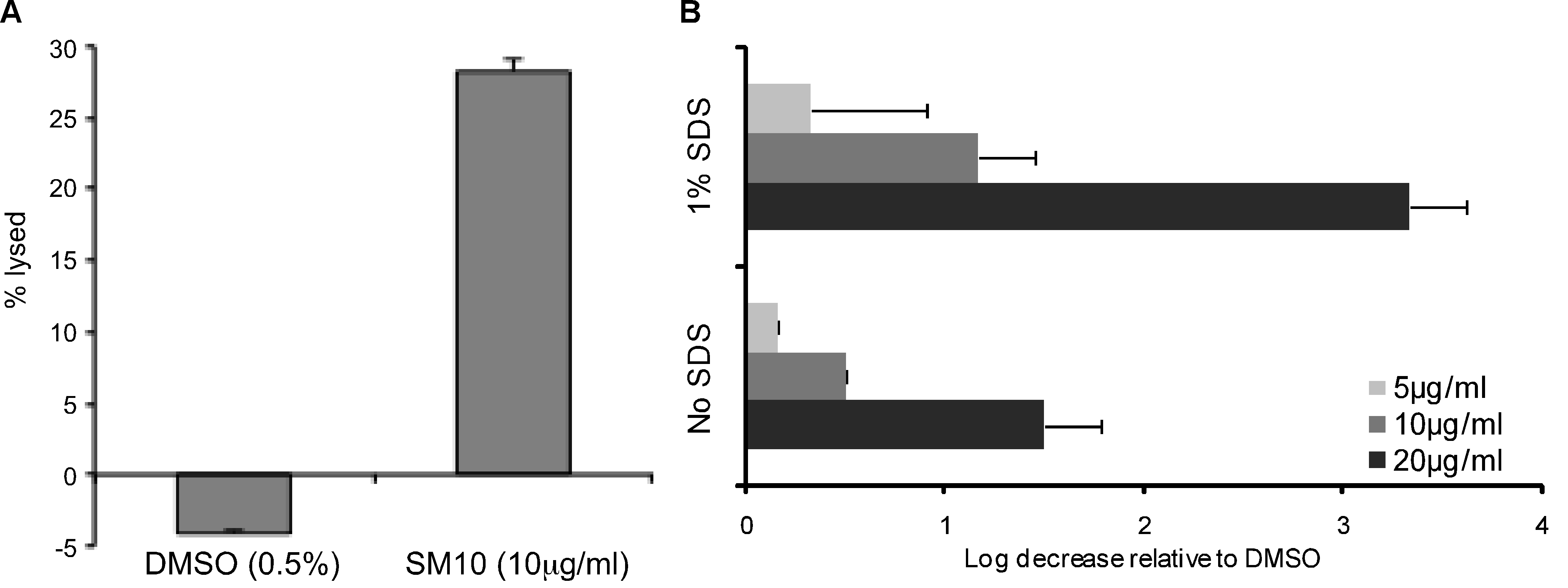
**

Supplement: Figure S3 — SM10 increased bacterial lysis and reduced viability of MG1655 in the presence of SDS. All cultures were grown in MHB. (A) E. coli MG1655 cells were incubated for 1 hr at 37°C in the presence of 10 µg/ml SM10 or DMSO. (B) Effect of SM10 on bacterial viability in conjunction with 1% SDS treatment. SDS (1%) was added to MG1655 cultures for 3 hours and the viability of the cultures was tested. (DOCX) [file pone.0044896.s003.docx]

**
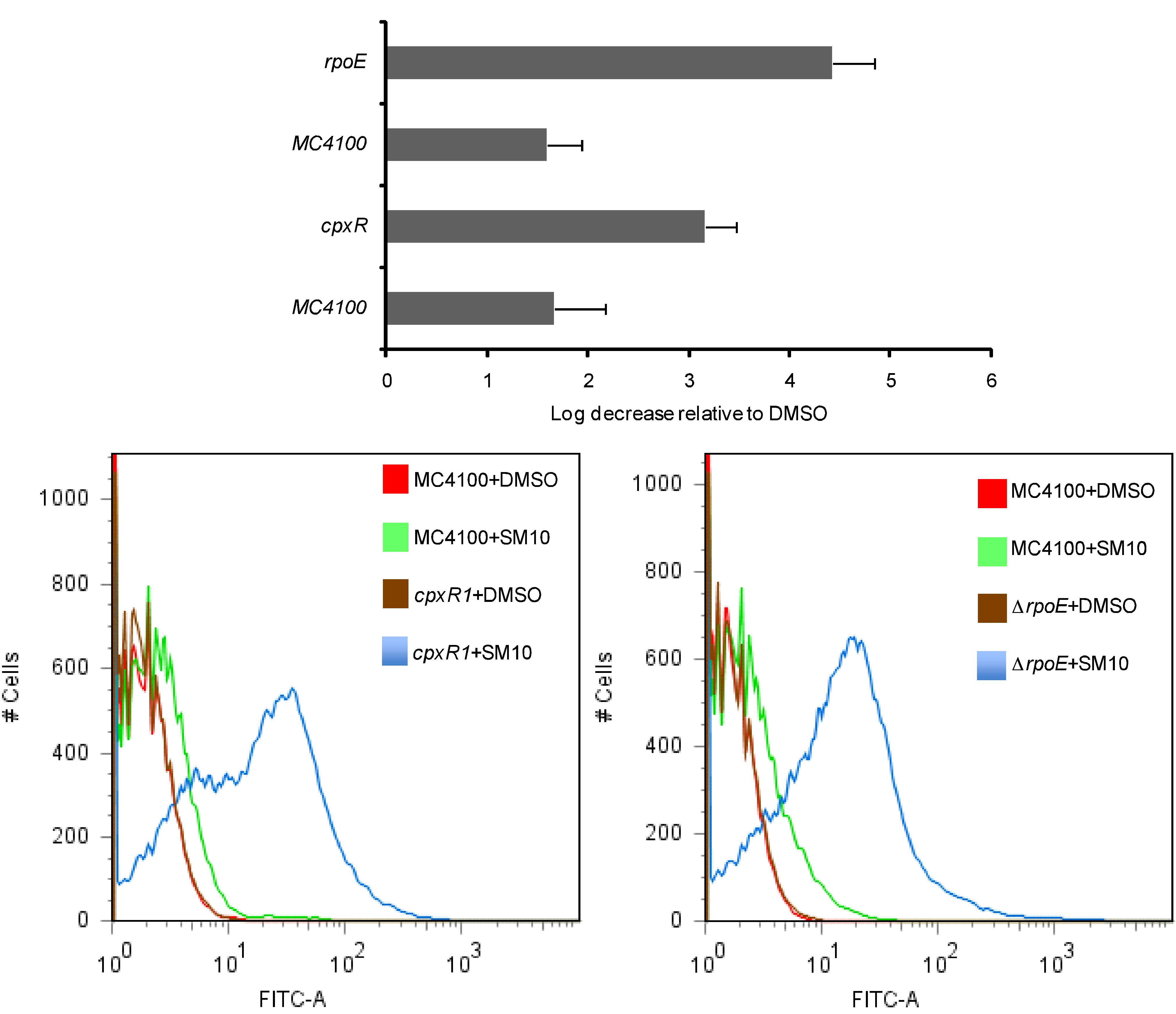
**

Supplement: Figure S4 — CpxR- and RpoE-deficient strains are hypersensitive to SM10. MC4100, cpxR1 and an ΔrpoE (suppressed by a mutation in ydcQ) strains were incubated in MHB at 37°C or 30°C, respectively, in the presence of DMSO or 10 µg/ml SM10. Cell viability relative to DMSO treatment was measured after 3 hours. (DOCX) [file pone.0044896.s004.docx]

**
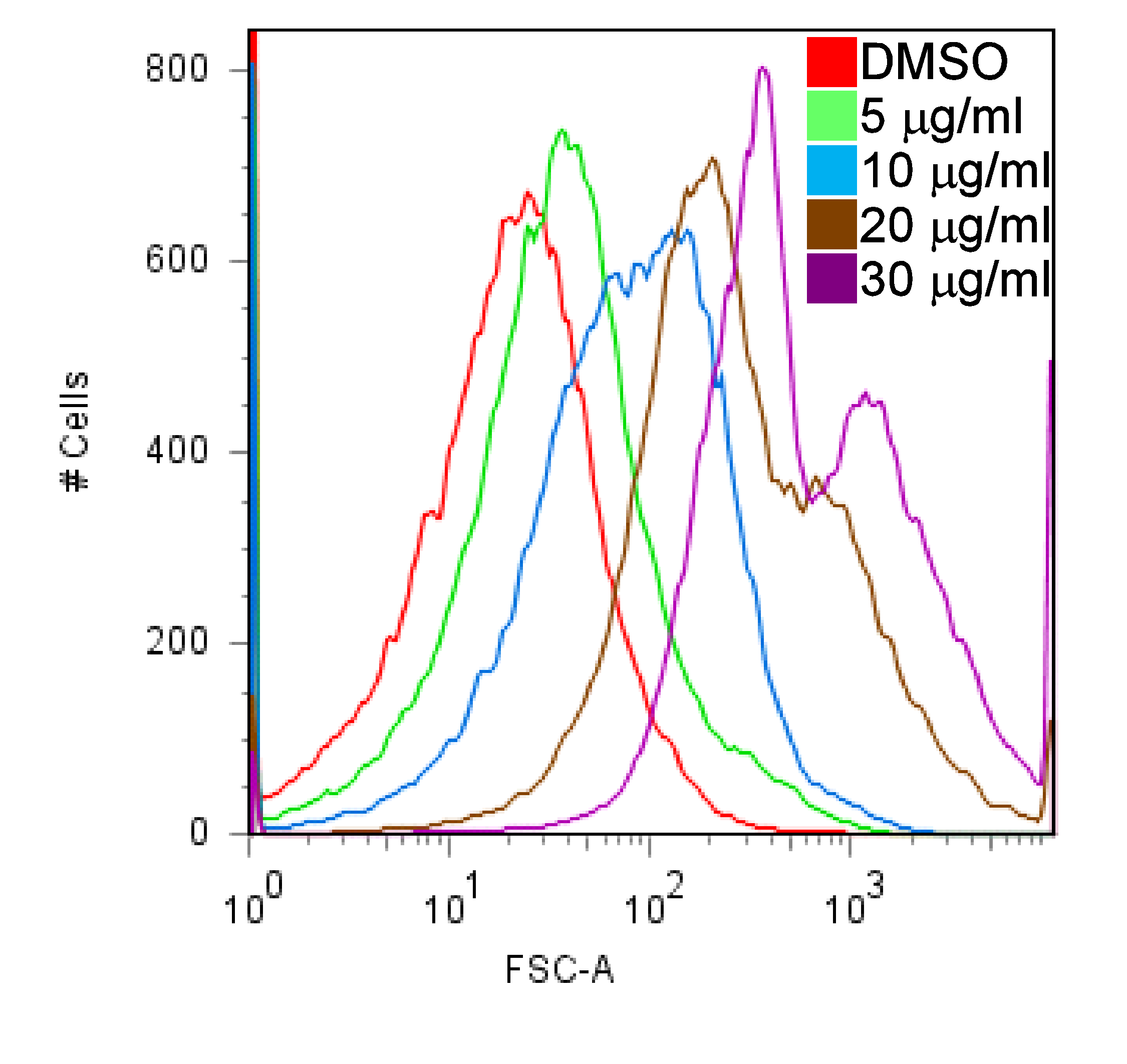
**

Supplement: Figure S5 — SM10 treatment caused filamentation, detected using flow cytometry. E. coli MG1655 incubated with the indicated concentrations of SM10 for 3 hours. The differences in the cell size parameter, FSC, are presented from the TUNEL experiment whose results are summarized in Table 3. (DOCX) [file pone.0044896.s005.docx]

**
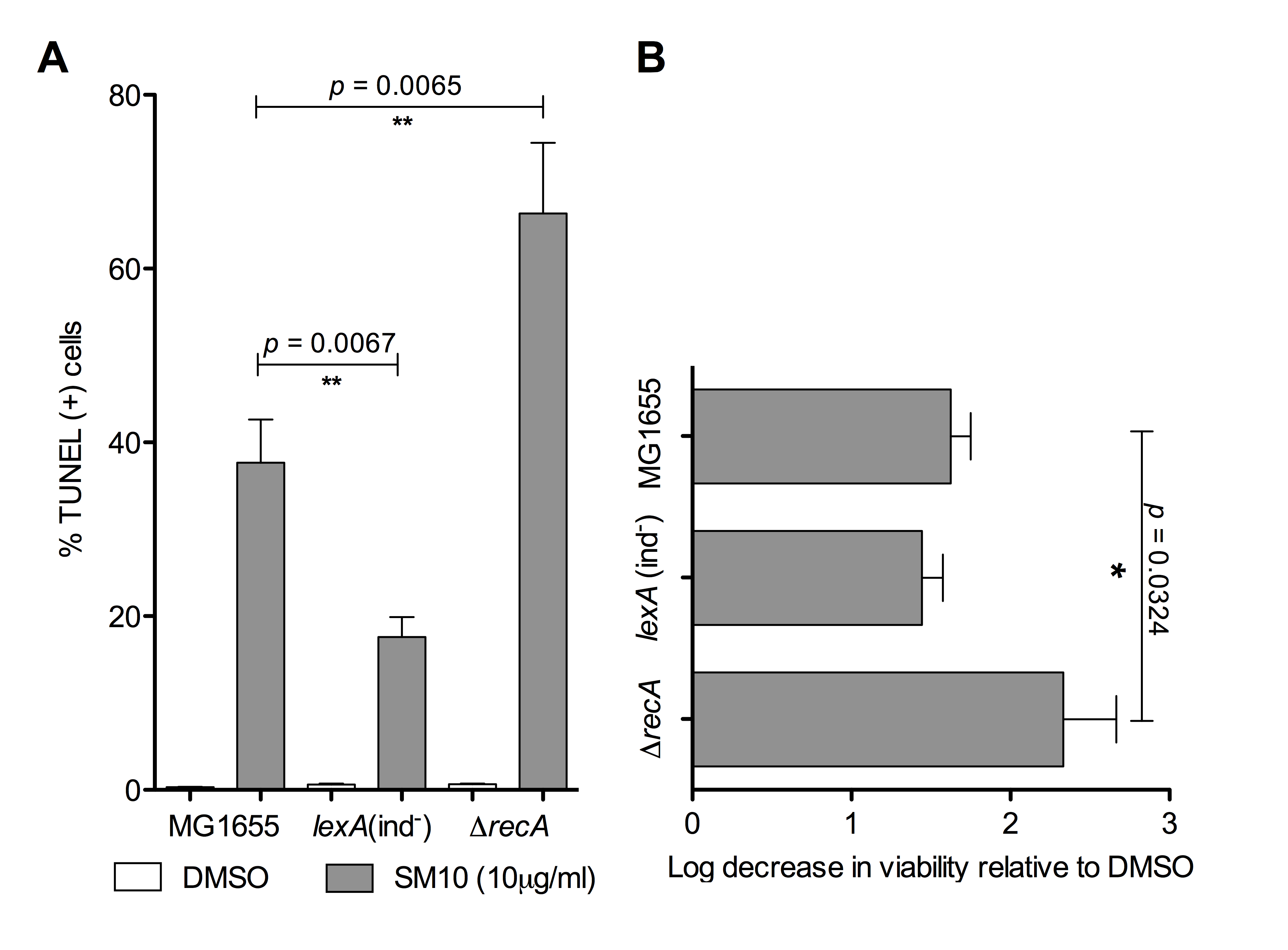
**

Supplement: Figure S6 — Results of TUNEL and viability assays performed in parallel on SOS deficient strains treated in exponential phase with DMSO or SM10 for 3 hours. A. TUNEL assay results. The strains specified were treated with DMSO or SM10 and DNA breaks were measured using the TUNEL assay (Methods). B. Viability assay results. Cultures were diluted, plated and the log decrease in viable cells calculated relative to DMSO treated cells. Data from 3 independent cultures per strain are shown. (DOCX) [file pone.0044896.s006.docx]

**
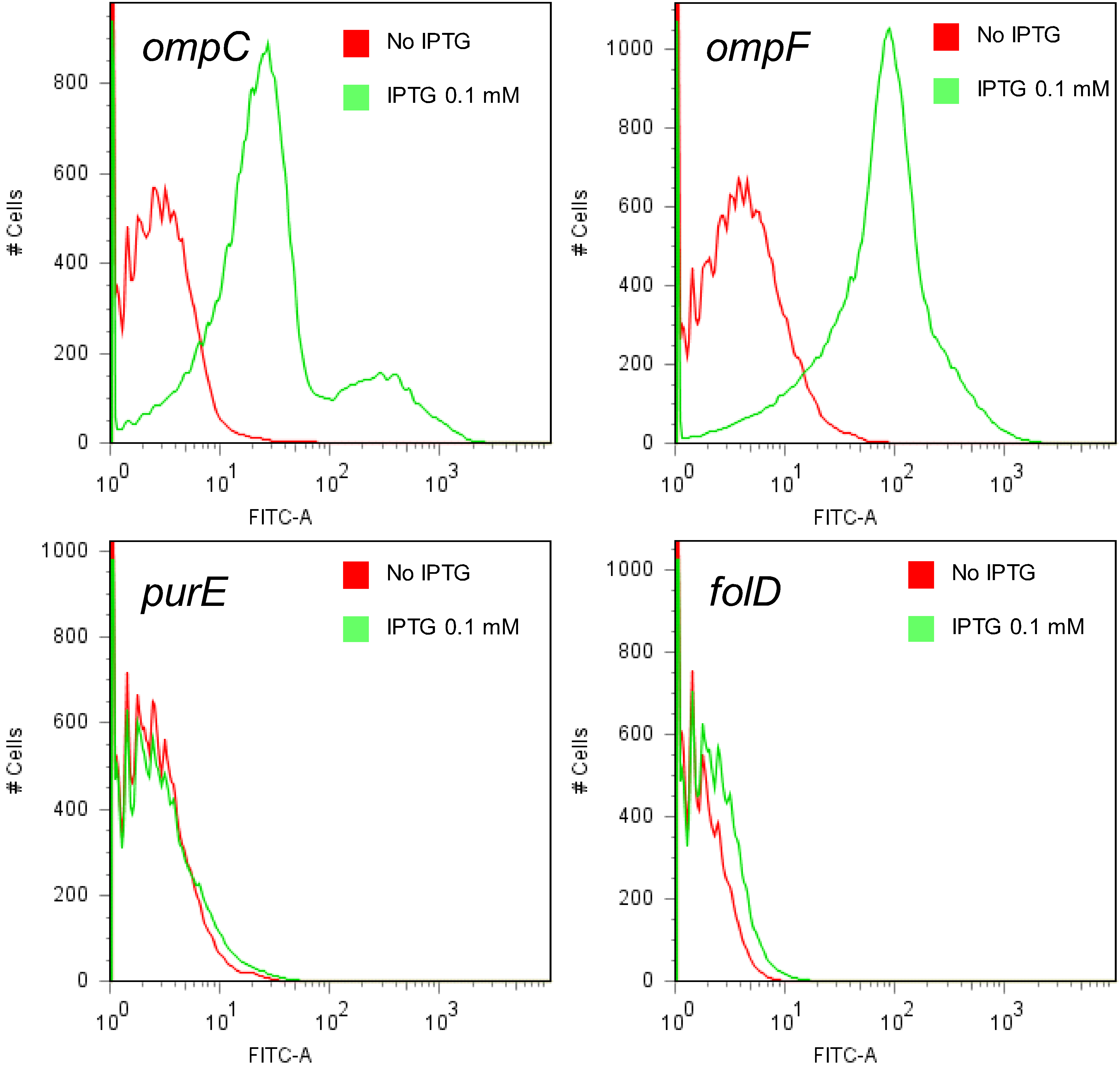
**

Supplement: Figure S7 — Overexpression of OmpC or OmpF causes an increase in free 3′OH DNA ends. Representative histograms are shown from TUNEL assays of MG1655 carrying high copy number plasmids encoding porins (OmpC or OmpF) or cytosolic proteins (PurE or FolD). Cells were incubated for 3 hr in the absence or in the presence of 0.1 mM IPTG. The TUNEL assay results were quantified using flow cytometry. (DOCX) [file pone.0044896.s007.docx]
