# Supplementary material for: Similarities between Exogenously- and Endogenously-Induced Envelope Stress: The Effects of a New Antibacterial Molecule, TPI1609-10
Source: PLoS One. 2012 Oct 11;7(10):e44896. doi: 10.1371/journal.pone.0044896 (PMC3469575; doi:10.1371/journal.pone.0044896)
Supplement: Figure S2 — Effect of SM10 treatment on metabolite incorporation in exponentially growing E.coli MG1655. A. Incorporation of 3H-thymidine to monitor DNA synthesis. B. Incorporation of 3H-uridine to monitor RNA synthesis. C. Incorporation of 14C-acetate to monitor phospholipid synthesis. D. Incorporation of 3H-leucine to monitor protein synthesis. For the experiments shown in panels A, B and C, cells were grown in MHB; for the experiment shown in panel D, cells were grown in MOPS medium supplemented with 0.2% glucose. The results are expressed as % incorporation, where measured incorporated cpm in SM10-treated cells were normalized to cpm incorporated in DMSO-treated cells. (DOC) [file pone.0044896.s002.doc]

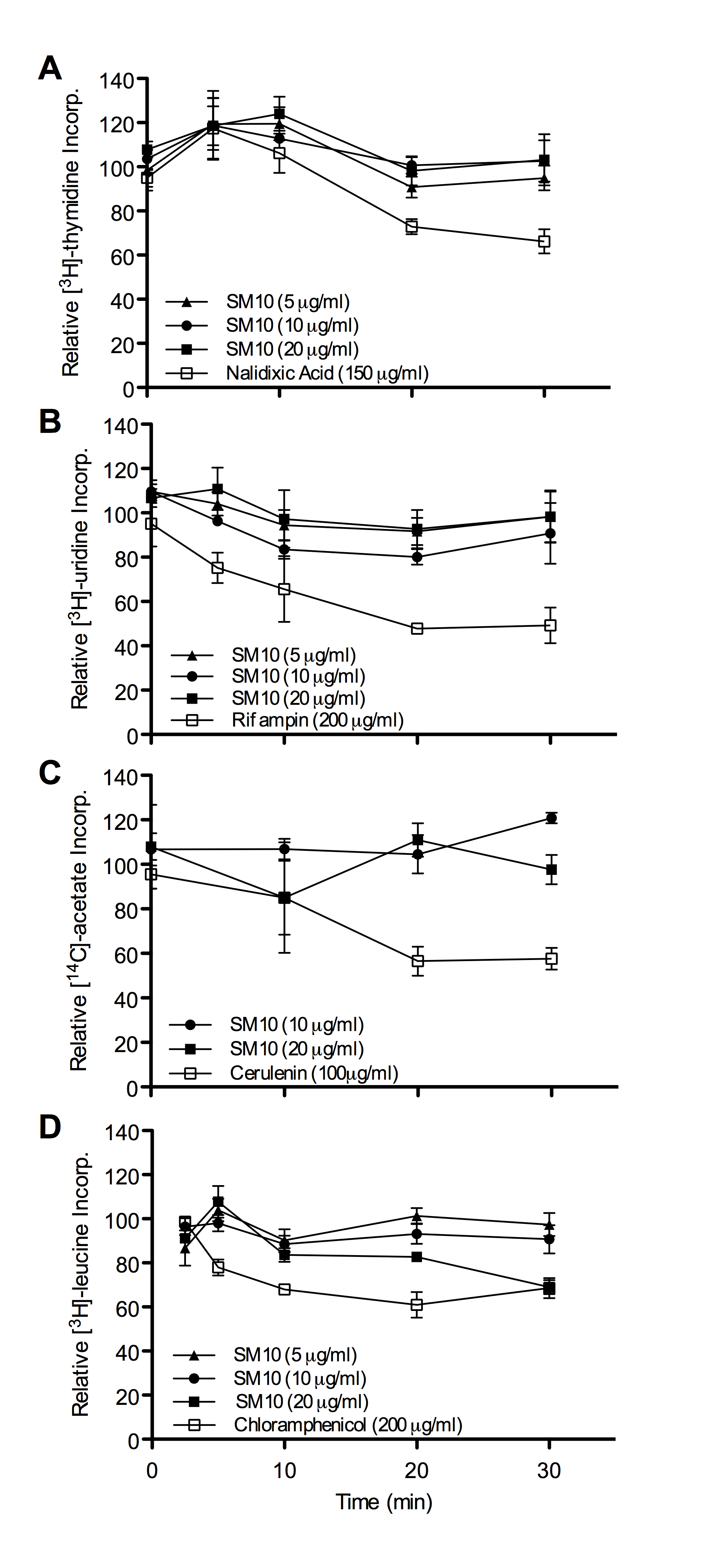


**Figure S2.** Effect of SM10 treatment on metabolite incorporation in exponentially growing *E.coli* MG1655. **A.** Incorporation of ^3^H-thymidine to monitor DNA synthesis. **B.** Incorporation of ^3^H-uridine to monitor RNA synthesis. **C.** Incorporation of ^14^C-acetate to monitor phospholipid synthesis. **D.** Incorporation of ^3^H-leucine to monitor protein synthesis. For the experiments shown in panels A, B and C, cells were grown in MHB; for the experiment shown in panel D, cells were grown in MOPS medium supplemented with 0.2% glucose. The results are expressed as % incorporation, where measured incorporated cpm in SM10-treated cells were normalized to cpm incorporated in DMSO-treated cells.
