# Supplementary material for: Similarities between Exogenously- and Endogenously-Induced Envelope Stress: The Effects of a New Antibacterial Molecule, TPI1609-10
Source: PLoS One. 2012 Oct 11;7(10):e44896. doi: 10.1371/journal.pone.0044896 (PMC3469575; doi:10.1371/journal.pone.0044896)
Supplement: Table S1 — TUNEL assay of SM10-treated Δ arcA cells or wild type isogenic cells. (DOCX) [file pone.0044896.s008.docx]

**Table S1.** TUNEL assay of SM10-treated Δ*arcA* cells or

wild type isogenic cells

| **Strain** | **% TUNEL^+^ cells^a^** | |
| --- | --- | --- |
|  | - SM10 | + SM10 |
| BW25113 | 1.1, 4.1 | 21.7, 26.4 |
| BW25113 Δ*arcA* (*frt*-Kan^R^-*frt*) | 0.1, 2.9 | 47.2, 83 |

**^a^**The results of two independent cultures are shown.
